# Temperature sensitivity of the mineral permafrost feedback at the continental scale

**DOI:** 10.1126/sciadv.adq4893

**Published:** 2024-10-09

**Authors:** Ella V. Walsh, Robert G. Hilton, Suzanne E. Tank, Edwin Amos

**Affiliations:** ^1^ Department of Earth Sciences, University of Oxford, Oxford OX1 3AN, UK.; ^2^ Department of Biological Sciences, University of Alberta, Edmonton, AB T6G 2H5, Canada.; ^3^ Aurora Research Institute, Western Arctic Research Center, Inuvik, NT X0E 0T0, Canada.

## Abstract

Oxidative weathering of sulfide minerals in sedimentary rocks releases carbon dioxide (CO_2_) into the atmosphere. In permafrost zones, this could be a positive feedback on climate change if it increases with warming, yet sulfide oxidation rates and their temperature response remain unknown over large spatial and temporal scales. We analyze a 60-year sulfate concentration dataset from catchments across the Mackenzie River Basin. Sulfate fluxes increased by 45% in the mainstem with 2.3°C of warming, and the temperature sensitivity suggests that continental-scale CO_2_ fluxes could double by 2100. The largest increases occur in catchments with geomorphic settings which act to rapidly expose rocks through physical weathering and thermokarst processes. Comparisons with a weathering model suggest that warming can increase reaction rates, and changes in the exposure of minerals with warming are also required. Future warming across vast Arctic landscapes could further increase sulfide oxidation rates and affect regional carbon cycle budgets.

## Teaser

Large Arctic catchment sulfide oxidation and CO_2_ release increased by 45%, representing positive feedback on warming.

## INTRODUCTION

Earth’s climate is moderated by the carbon cycle and fluctuations in long-lived atmospheric greenhouse gas concentrations (e.g., carbon dioxide, CO_2_). Oxidative weathering plays an important role in releasing carbon from geological sedimentary stores (*1*). A key pathway involves the production of sulfuric acid (H_2_SO_4_) through the oxidation of sulfide minerals (e.g., pyrite, FeS_2_). This acid can react with carbonate minerals to release CO_2_ to the atmosphere immediately [Eq. 1; (*2*, *3*)] or through interaction with the bicarbonate pool (*4*)

$${\text{CaCO}}_{3}+H_{2}{\text{SO}}_{4}\to {\text{CO}}_{2}\left(g\right)+H_{2}O+{\text{Ca}}^{2+}+{{\text{SO}}_{4}}^{2-}$$ (1)

Sulfide oxidation can affect CO_2_ concentrations in the ocean-atmosphere system (*5*) and has been linked to subsequent carbon and oxygen cycle feedbacks (*6*). The associated carbonate dissolution is invoked to play a central role in the CO_2_ balance of weathering and erosion across landscapes (*2*, *7*). These reactions are recognized in terms of contemporary and future fluxes of carbon, notably via the mineral permafrost carbon cycle feedback (*8*, *9*). It is also an important and understudied process in the longer-term carbon cycle on glacial-interglacial (*10*) to Cenozoic timescales (*5*). Riverine fluxes of dissolved sulfate (SO_4_^2−^) sourced from sulfide oxidation provide a tracer of landscape-scale processes (*11*). Current estimates from global SO_4_^2−^ flux data suggest a release of ~30 to 40 million tonnes of carbon per year (Mt C year^−1^) from oxidative weathering of pyrite (*4*, *8*).

Sulfide oxidation rates are changing over decadal timescales (*9*), but we know very little about how they will change over the next 100 years. Recent studies have shown that sulfide oxidation is sensitive to climate change, potentially acting as an amplifying feedback on warming. First, SO_4_^2−^ export from small catchments in high altitude settings (*9*) provides evidence of sulfide oxidation increasing exponentially with rising air temperature. Second, at the scale of rock outcrops (*3*, *12*), direct measurements of CO_2_ release during sulfide oxidation show an exponential increase with air temperature. These local-scale measurements suggest that the flux may double for a temperature rise of 10°C (*3*). In addition, physical weathering processes have been identified as being important for sulfide oxidation (*13*). Frost cracking is one such climate-sensitive weathering process that can produce microfractures and generate large amounts of reactive mineral surface area (*14*–*17*).

Surface air temperatures in the Arctic are increasing nearly four times faster than the global average (*18*), leading to warming permafrost, thickening the active layer (*19*, *20*), and changing hydrological and biogeochemical processes (*21*). High-latitude regions represent sensitive stores of carbon and the impact of thaw on CO_2_ release from soil organic carbon decomposition may be substantial (*22*). Where ground ice is present, permafrost thaw can result in landscape collapse as thermokarst (*23*, *24*). Recent work on retrogressive thaw slumps in thermokarst landscapes has highlighted the potential for a mineral permafrost CO_2_ leak in the Arctic (*25*). Reactive sulfide minerals in glacial tills weather rapidly following exposure to erosion and are as responsive as organic matter degradation during thaw (*25*). Thermokarst could therefore play a critical role in the response of sulfide oxidation to Arctic warming (*23*, *25*–*27*). In addition, active layer thickening can affect hydrological flow paths and the depth of the unsaturated zone (*28*, *29*), increasing access to reactive sulfide minerals. However, the temperature sensitivity of the oxidative CO_2_ release has not been established at large spatial scales, and the underlying geomorphic, hydrological, and biogeochemical drivers remain obscured. It is not yet possible to account for this CO_2_ release and its drivers in the carbon cycle budgets of the future Arctic (*30*).

**Fig. 1.**
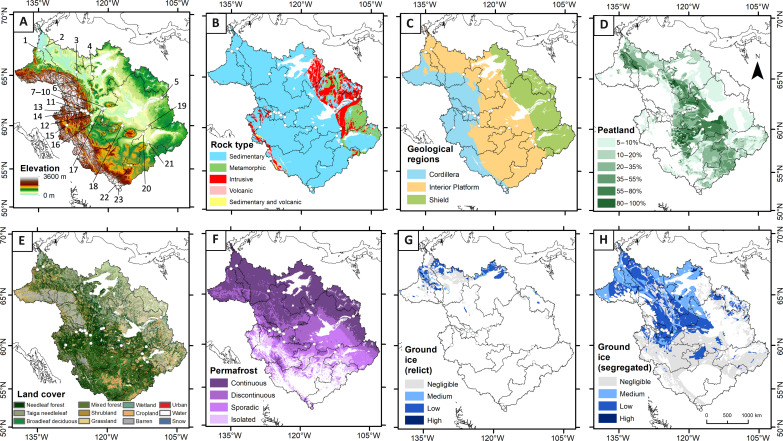
Mackenzie catchment characteristics. (**A**) Elevation (Shuttle Radar Topography Mission digital elevation model) with sites labeled (see table S1 for site names and data availability). (**B** and **C**) Bedrock lithology (*45*). (**D**) Peatland (*41*). (**E**) Land cover (*42*). (**F**) Permafrost (*43*). (**G**) Relict ground ice cover (*44*). (**H**) Segregated ground ice cover (*44*).

Within this high-latitude region, the Mackenzie River Basin (MRB), Canada, is a major source of freshwater (*31*), sediment (*32*), solutes (*33*, *34*), and organic carbon (*35*) to the Arctic Ocean. Sulfides within shales are colocated with carbonates in the MRB, particularly in the Rocky and Mackenzie Mountains (*33*, *36*), and the basin is dominated by sedimentary rocks (68.3%) found in the Cordillera and Interior Platform (Fig. 1). Isotopic tracing of sulfate in the Mackenzie (*2*, *26*, *37*) has enabled the contribution of sulfide oxidation to dissolved SO_4_^2−^ fluxes to be quantified, and SO_4_^2−^ flux increases have been previously noted in the Mackenzie mainstem, Liard, and Peel rivers (*25*, *26*). However, analysis of a subset of catchment areas means that the spatial and temporal patterns of change and the geomorphic controls and impact of a warming climate on sulfide oxidation have not been assessed. The associated CO_2_ release could affect the future regional carbon budget, but it has not been included in geochemical modeling studies to date (*38*). Previous model assessments suggested a 50% increase in carbonate weathering until the end of the century could act as a growing natural CO_2_ sink in the Mackenzie River system (*38*).

Here, we quantify the temperature sensitivity of sulfide oxidation and the main drivers by investigating a 60-year dataset of water geochemistry from catchments across the MRB. Water hydrochemical measurements from 1960 to 2020 were obtained by Environment and Climate Change Canada (*39*) and daily discharge data by the Water Survey of Canada (*40*), covering 23 major and minor catchments of the MRB (Fig. 1A). This dataset captures the geochemical signature of high and low flow regimes. In addition, we establish the contemporary SO_4_^2−^ export from the Tsiigèhnjik (Arctic Red) catchment using an annual time series. We quantify spatial and temporal air temperature changes at the catchment scale, alongside metrics relevant to physical and chemical weathering processes (bare rock cover, slope, permafrost extent, ground ice extent, peatland cover, and lithology) (*41*–*45*). By doing so, we uncover catchment-specific links between SO_4_^2−^ fluxes and temperature change. A weathering model suggests the need for a combined response of reaction kinetics and mineral surface area generation processes with climate change. By establishing the temperature dependency of sulfide oxidation at the continental scale, we predict the magnitude of CO_2_ fluxes under future scenarios of warming and demonstrate that this is a positive feedback on climate change that needs to be accounted for in future carbon budgets.

## RESULTS

### Multidecadal increase in river SO_4_^2−^ concentration and flux

**Fig. 2.**
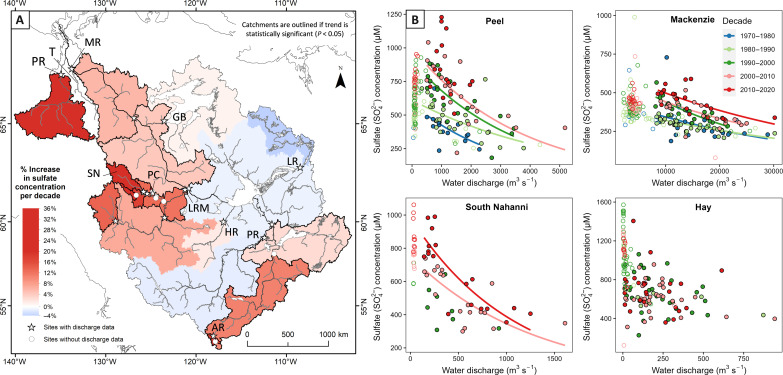
Mackenzie SO_4_^2−^ concentration change over time. (**A**) SO_4_^2−^ concentration change (%) per decade by catchment. Catchments are outlined with a bold black line if the trend of SO_4_^2−^ concentration with time is statistically significant (*P* < 0.05). Catchments discussed in the text are labeled. MR, Mackenzie River above Tsiigehtchic; T, Tsiigèhnjik; PR, Peel River; GB, Great Bear; LR, Lockhart River; LRM, Liard River near mouth; PC, Prairie Creek near mouth; SN, South Nahanni above Virginia Falls; HR, Hay River; PR, Peace River at Peace Point; AR, Athabasca River at HWY 16. (**B**) Relationship between measured SO_4_^2−^ concentration and water discharge, colored by decade to detail the decadal change in SO_4_^2−^ concentration across all hydrological pools for the Peel River, Mackenzie River above Tsiigehtchic, South Nahanni above Virginia Falls, and Hay River. Points detail sampling through ice (open points) and ice-free time steps (closed points). Power law fits are drawn through ice-free sampling data when statistically significant (*P* < 0.05).

The Mackenzie mainstem site (above Tsiigehtchic; site NW10LA0003) records a significant increase in catchment-wide SO_4_^2−^ concentration by 6.1 ± 1.2% decade^−1^ (1971–2019) (Fig. 2). Annual SO_4_^2−^ concentrations increased in datasets ranging from half a century to less than a decade (fig. S1), increasing by 20.4 ± 1.7% decade^−1^ and 20.9 ± 2.4% decade^−1^ in the Peel (1973–2017) and South Nahanni catchments (1994–2018), respectively, and up to 36.4 ± 4.4% decade^−1^ in the Prairie Creek (2005–2018), which is a small watershed within the Canadian Cordillera. In contrast, no significant trends in SO_4_^2−^ concentrations were found in the Great Bear, Lockhart, Peace, and Hay catchments.

Dissolved ion concentrations do not directly translate to changes in weathering flux, with water discharge being a key variable. However, we note that all catchments show no significant change in annual water discharge over the study period (fig. S2). To help account for hydrological variability, daily SO_4_^2−^ flux estimates were quantified using the Weighted Regressions on Time, Discharge, and Season (WRTDS) model (see Materials and Methods) from paired SO_4_^2−^ concentration and water discharge measurements, combined with daily water discharge. These data are available for 14 catchments (table S1). Over the monitoring period, mean annual SO_4_^2−^ fluxes increased by 45 and 167% in the Mackenzie mainstem (1972–2019) and Peel River (1973–2017), respectively (table S2). This is an increase from 87.2 to 127.2 Gmol year^−1^ in the Mackenzie mainstem and 7.3 to 19.4 Gmol year^−1^ in the Peel. Combining the data from these two sites with the Tsiigèhnjik (see Materials and Methods), the total SO_4_^2−^ flux from the MRB is currently 151 Gmol year^−1^.

**Fig. 3.**
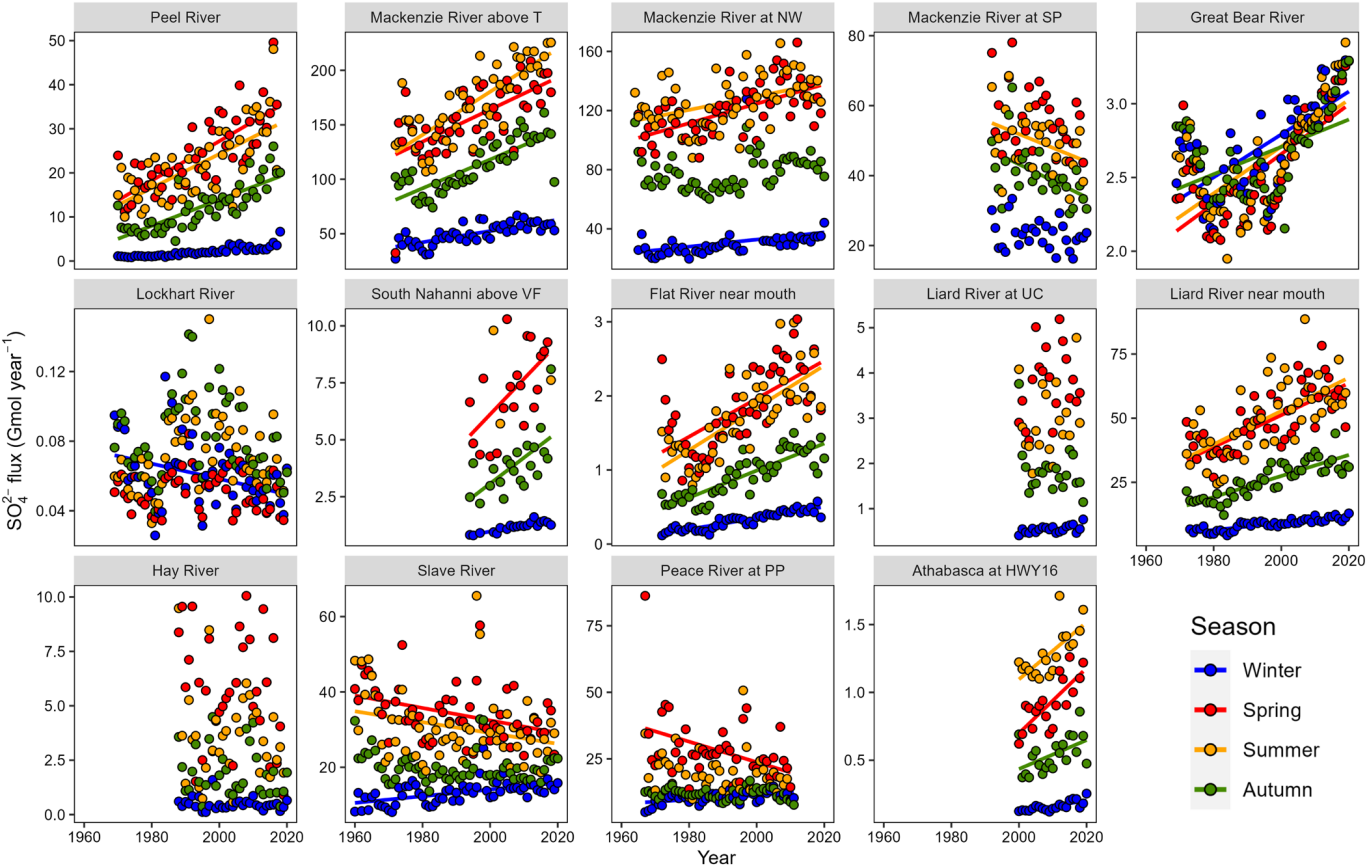
Trends in a seasonal average of WRTDS (Weighted Regressions on Time, Discharge, and Season) modeled SO_4_^2−^ flux by the river. Significant regression lines have been plotted (*P* < 0.05). Winter: December to April; Spring: May to June; Summer: July to August; Autumn: September to November. See table S1 for locations and site codes.

We account for the sources of SO_4_^2−^ using published data (*2*) and independent observations of SO_4_^2−^ production and export (see the Supplementary Materials). Having done so, the corresponding sulfide oxidation flux is estimated to be 129 Gmol year^−1^, which is similar to findings from a more data-limited study (*2*). Most rivers display a seasonal pattern in discharge and SO_4_^2−^ concentration and flux (Fig. 3 and fig. S1) and show dilutional concentration-discharge patterns (Fig. 2B). When the concentration-discharge data are subset by decade, we see increasing concentrations across all periods of open water and see no shift in the range of discharge values (Fig. 2B).

### Temperature changes and SO_4_^2−^ fluxes

**Fig. 4.**
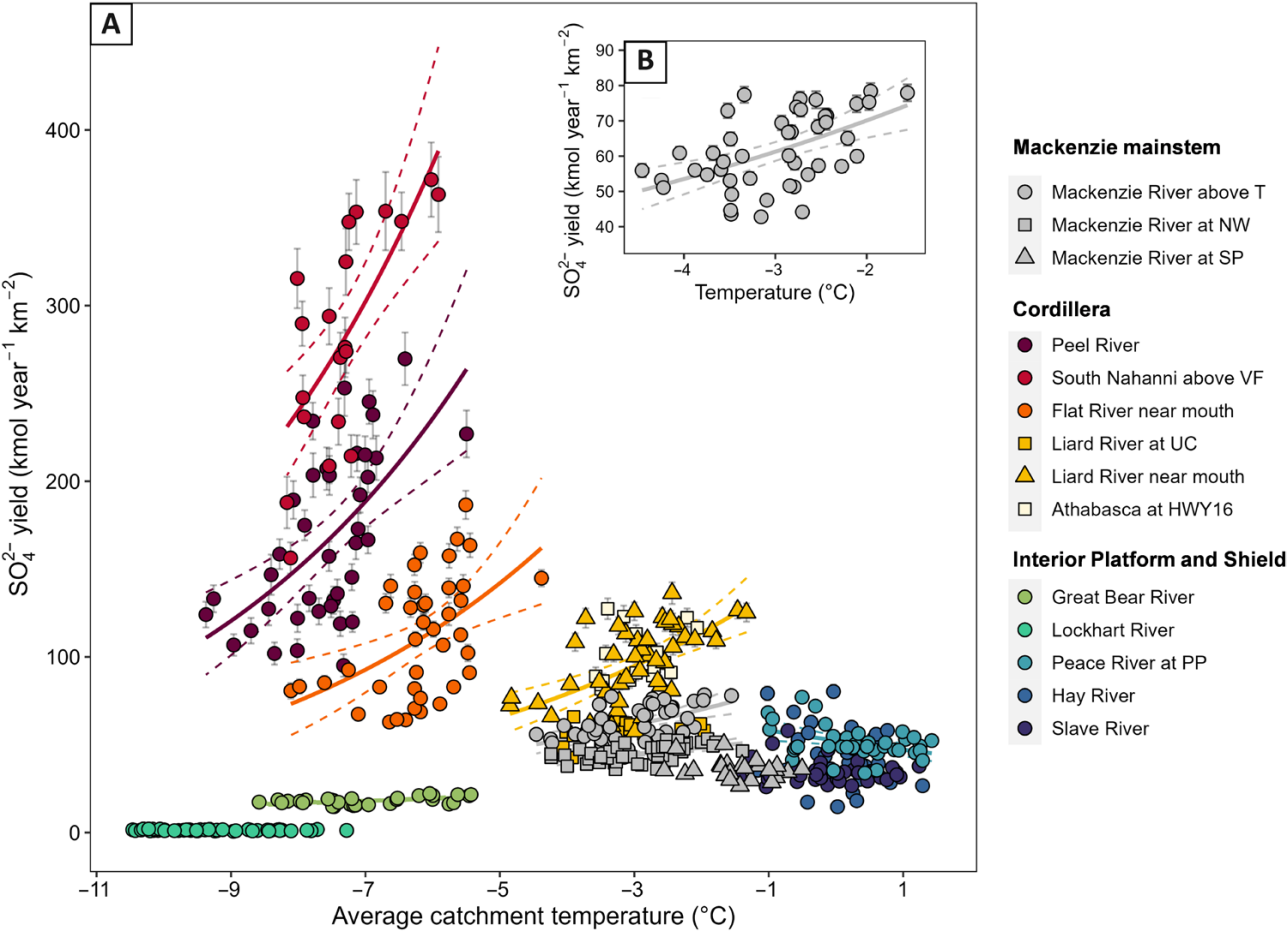
Temperature sensitivity of riverine SO_4_^2−^ yield (flux normalized to catchment area). (**A**) All rivers with WRTDS (Weighted Regressions on Time, Discharge, and Season) modeled flux data and (**B**) close-up of the Mackenzie above Tsiigehtchic dataset. Exponential function (solid lines) and 95% confidence interval (dashed lines) are drawn for rivers where the relationship is statistically significant (*P* < 0.05). Error bars show the standard error on annual SO_4_^2−^ yield estimates when larger than the point size. See table S1 for locations and site codes.

Changing SO_4_^2−^ fluxes of MRB catchments correlate positively with catchment-averaged air temperatures (Fig. 4). The relationships can be fitted with an exponential model consistent with first-order reaction kinetics (see Materials and Methods). An exponential function of temperature and sulfide oxidation has been recently reported for small alpine catchments (*9*) and from in situ measurements at the rock outcrop scale (*3*). The model fit returns the value *F*_0_, which is the SO_4_^2−^ flux at 0°C, and an exponential growth rate parameter α of the relationship (in reciprocal degree Celsius). To account for controls of hydrology on interannual variability, we undertake this analysis on both SO_4_^2−^ concentration (fig. S3) and SO_4_^2−^ yield (flux normalized to catchment area) (Fig. 4) and find that trends are consistent. We also find no statistically significant relationship between mean annual precipitation and SO_4_^2−^ yield (see Materials and Methods).

Baseline annual SO_4_^2−^ yields and concentrations (Fig. 4 and fig. S3) and *F*_0_ values (table S2) vary between catchments, with yields ranging from 1.4 kM year^−1^ km^−2^ (Lockhart River) to 283.4 kM year^−1^ km^−2^ (South Nahanni). The growth rate parameter α describes the sensitivity of Mackenzie riverine SO_4_^2−^ export to air temperature. Across catchments, this value ranges from 0.04 ± 0.41°C^–1^ (Peace River: *P* < 0.05) to 0.26 ± 0.07°C^–1^ (South Nahanni River, *P* < 0.001) for significant fits (table S2). Temperature-specific changes in Mackenzie mainstem yields are described by an α of 0.12 ± 0.04°C^–1^. This can be used to calculate a *Q*_10_ value that records the factor by which the yield increases over a 10°C increase in temperature (see Materials and Methods), returning a *Q*_10_ value of 3.6 ± 1.0 (*r*^2^ = 0.24, *P* < 0.001, *n* = 34). *Q*_10_ values for Mackenzie catchments reach up to 9.15 ± 2.15 (*P* < 0.001, *n* = 39) in the Peel River and 13.3 ± 3.4 (*P* < 0.001, *n* = 20) in the South Nahanni River. Nonsignificant and comparatively low *Q*_10_ values are found for the Lockhart and Great Bear catchments.

We note that any temperature control on reaction kinetics defined by the growth rate parameter α is “apparent” (*46*), as it integrates the intrinsic sensitivity of sulfide oxidation to temperature and catchment scale geomorphic and hydrological changes. The Mackenzie *Q*_10_ value of 3.6 ± 1.0 is comparable to laboratory oxidation experiments of crushed pyrite substrate under fully oxic conditions [2 to 3 (*47*)] and it is slightly higher than an outcrop scale estimate for CO_2_ generation from carbonate dissolution associated with sulfide oxidation [1.7 ± 0.3; (*3*)]. The apparent *Q*_10_ value for sulfide oxidation is similar to other, more well-studied, carbon-cycle climate feedbacks such as soil organic matter respiration, which has a *Q*_10_ value of 3.0 ± 1.1 for a global soil compilation (*48*) and a *Q*_10_ value of 2.4 ± 0.3 in a soil warming experiment (*49*).

### Geospatial analysis of SO_4_^2−^ fluxes and temperature response

**Fig. 5.**
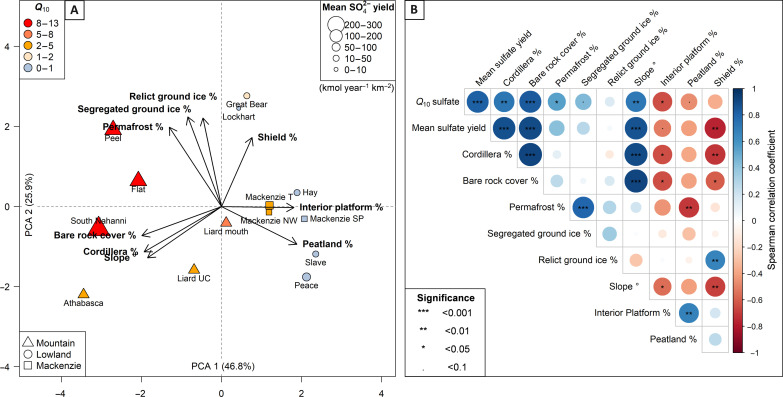
Assessment of catchment controls on the SO_4_^2−^ yield and temperature sensitivity (*Q*_10_). (**A**) Principal components analysis (PCA) of catchment characteristics (Fig. 1). Points are colored by the *Q*_10_ value (for SO_4_^2−^ yield data) and the size of the points represents the mean catchment SO_4_^2−^ yield. The angle between vectors details the correlation between different components: 0°, 90°, and 180° correspond to correlation, no correlation, and inverse correlation, respectively, where sites near each other have similar catchment characteristics. (**B**) Correlation matrix of catchment characteristics and SO_4_^2−^ data. Shading reflects the correlation coefficient and the significance of the relationship is demarcated within each point.

To investigate possible landscape-scale controls on sulfide oxidation in the MRB, we quantified catchment characteristics relevant to weathering (Fig. 1). The MRB is geologically and geomorphologically diverse, and principal components analysis (PCA) shows that catchment characteristics covary (Fig. 5A). Rivers such as the Peel, South Nahanni, and Flat drain the Cordillera lithology and these catchments are characterized by high slope settings, high bare rock cover, and high ground ice and permafrost extents (Fig. 5A). In contrast, the Lockhart and Great Bear are sourced from the Shield where sedimentary sulfides are rare, and the Slave, Peace, and Mackenzie have high peatland cover and drain the central plains of the Interior Platform.

We use the location of rivers within the PCA plot to examine the relationship between catchment controls and metrics relating to sulfide oxidation. The combined influence of multiple catchment parameters explains the variability in the SO_4_^2−^ yield and temperature response (*Q*_10_) data (Fig. 5B). SO_4_^2−^ yields and *Q*_10_ values are highest in catchments draining the Cordillera and lowest in catchments draining the Shield lithologies. Bare rock cover and average slope angle are positively correlated with baseline SO_4_^2−^ yields. Permafrost extent, ground ice extent, and bare rock cover are positively correlated with *Q*_10_ values. Peatland cover is negatively correlated with both yield and *Q*_10_. Together, these attributes point toward an important role of physical weathering and erosion in setting the apparent temperature sensitivity of sulfide oxidation.

## DISCUSSION

### Geomorphic and hydrological controls on SO_4_^2−^ flux

A number of catchments contribute disproportionately relative to their area to the overall SO_4_^2−^ export of the MRB. The South Nahanni and Prairie Creek catchments each contribute 4.8 times the SO_4_^2−^ flux per unit expected relative to the MRB mean, highlighting the importance of interrogating geochemical fluxes at the catchment scale. These high yields in part reflect a lithological control. Sulfides are present in rocks within the Mackenzie mountains (*36*, *50*) and rivers draining this region have high SO_4_^2−^ concentrations and yields (Figs. 3 and 5A and fig. S1). However, variability in catchment-averaged SO_4_^2−^ yields is linked to geomorphic controls (Fig. 5).

**Fig. 6.**
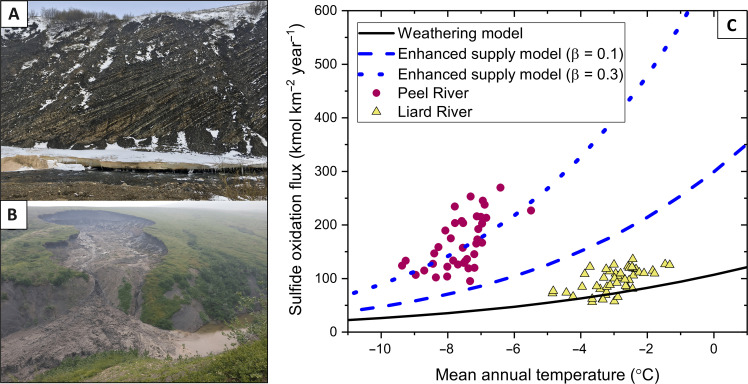
Enhanced supply of sulfide minerals to the weathering zone seen in landscapes and captured in a weathering model. (**A**) Landscape in the upper Peel River showing exposed sedimentary bedrock on steep slopes coupled to river channels, where physical weathering is producing abundant fresh material (photo credit: R.G.H.). (**B**) Thaw slump on the Peel Plateau, which exposes sulfide and carbonate minerals in glacial till to surface weathering reactions in lower slope regions with relict ice (photo credit: S.E.T.). (**C**) Weathering model outputs showing kinetically limited weathering rates (black line) and the enhanced supply model as a function of temperature, which can better explain the high-temperature sensitivity recorded in the mountainous Peel River catchment (data shown for comparison).

The highest SO_4_^2−^ yields are recorded in catchments that have geomorphic settings that expose rocks to oxygen in the weathering zone (Fig. 5B): high slope environments and catchments with substantial bare rock cover (Fig. 6A). Oxidation of pyrite occurs rapidly (*51*) and the rate is often limited by the availability of fresh material, increasing with erosion rate (*2*, *7*, *13*, *52*). In these high-elevation and high-latitude settings, physical weathering processes can moderate sediment generation (*17*), with frost cracking being an important mechanism (*14*, *53*, *54*). Between temperature windows of −3° to −8°C, frozen water in pores can efficiently lead to the generation of cracks, exposing fresh mineral surfaces and leading to the generation of sediment in regions that experience temperatures in this window. In contrast, sites with the highest peatland cover have lower SO_4_^2−^ yields (Fig. 5). This would be consistent with a reduction in oxygen delivery to rock minerals as overlying soils limit oxygen diffusion (*55*), while physical weathering rates are also reduced.

Thermokarst processes can also enhance sulfide oxidation rates, where permafrost and ground ice extent have a positive relationship with baseline SO_4_^2−^ yields (Fig. 5B). Excess ground ice can make landscapes susceptible to thermokarst processes (*23*, *24*). First, the melt of relict ice embedded in freshly deposited glacial tills can cause landscape mass movement (*24*). In the Peel Plateau, retrogressive thaw slumps (Fig. 6B) increase downstream SO_4_^2−^ concentrations through the exposure and rapid weathering of tills rich in unweathered sulfide and carbonate minerals (*25*, *56*). Second, segregated ice that forms at the base of the active layer can cause frost heave and ground subsidence. The thaw of segregated ice lenses can enable water percolation to deeper mineral soils (*57*, *58*). These geomorphic processes are likely to be important mechanisms in settings beyond the Peel River, where glacial margin landscapes are found across the western Canadian Arctic and thaw slump activity is extensive (*23*).

In addition to these geomorphic mechanisms, the changing thickness of the active layer and nature and pathways of hydrological flow paths are likely to play a role (*27*, *59*). Deepening of an active layer would allow physical weathering processes to operate deeper in the substrate, while thickening of the unsaturated zone would supply more O_2_ by gas diffusion. The removal of weathering products by connected subsurface flow paths will also help to sustain reactions (*60*). Together, these geomorphic and hydrological changes can explain the increases in sulfide oxidation found across these vast river catchments.

### Temperature sensitivity of sulfide oxidation

Our data show that sulfides in the Mackenzie catchments can be highly reactive, even at subzero mean annual air temperatures (Fig. 4). The basin average *Q*_10_ value of 3.6 ± 1.0 is similar to those derived from experimental and field-based measurements of sulfide oxidation (*3*, *47*). This temperature sensitivity is large in comparison to that of silicates (*61*). The range in *Q*_10_ values supports experimental studies that find high-temperature coefficients of sulfide oxidation at low temperatures (*48*, *62*). These experiments show *Q*_10_ values of around 10 for temperatures below −2°C and *Q*_10_ values of around 2 above this temperature (*62*). Freezing of pore waters presents a physical limitation on oxygen transport, but as temperatures warm, the potential for increases in sulfide oxidation is large (*62*).

The spatial variability in SO_4_^2−^ yield change with temperature (Fig. 5) indicates that the temperature response of sulfide oxidation is moderated by catchment attributes in a similar way to baseline SO_4_^2−^ yields. This suggests that the underlying geomorphic and hydrological drivers are linked to ongoing temperature change (*17*). The *Q*_10_ values we observe integrate both a direct temperature effect on the reaction kinetics and the effect of increasing substrate exposure with warming, for example, through physical weathering processes and active layer thickening. In steep mountain environments with bare rock cover, we find very high *Q*_10_ values in the Peel and South Nahanni catchments. There, geomorphic processes can rapidly expose rocks to weathering through mechanical fracturing of exposed rocks (Fig. 6A) and thermokarst processes (Fig. 6B) during permafrost thaw. In lower elevation settings, the nature of permafrost thaw is dependent on the distribution of ground ice (*27*) and thermokarst disturbances in incised ice-rich landscapes are highly vulnerable to warming (*24*, *63*). This is supported by a positive relationship between *Q*_10_ values and permafrost and ground ice extent (Fig. 5B). With rising temperatures, more extensive thermokarst processes have been observed across the Canadian Arctic (*64*), with retrogressive thaw slumps increasing in both in intensity and frequency (*65*). Land surface collapse in relict ice–rich settings rapidly increases the exposure of sulfides to the surface, and the thaw of segregated ice lenses enables greater interaction between water and deep soil horizons, both sustaining a supply of material to weather with warming.

To better constrain the coupled physical and biogeochemical drivers of the temperature sensitivity of sulfide oxidation, we explore the underlying processes using a weathering model (*52*, *66*, *67*). Recent work has shown that in subtropical and temperate mountains, sulfide oxidation is supply limited up to high erosion rates of >2000 tonnes km^−2^ year^−1^ (*52*). However, cooler-than-average air temperatures in the Mackenzie subcatchments mean that reactions are likely to be slower overall, and thus, kinetically limited reactions may be found at lower erosion rates. This is confirmed by a weathering model (see Materials and Methods) where we predict a temperature sensitivity of sulfide oxidation at erosion rates of 295 tonnes km^−2^ year^−1^ at mean annual temperatures typical of the MRB catchments (Fig. 6C). However, the weathering model cannot reproduce the very high-temperature sensitivities that we find in the Peel and South Nahanni catchments.

We modify the weathering model to consider the role of an enhanced supply of sulfides due to changes in temperature. Following discussion of underlying mechanisms, these could include (i) physical weathering processes which increase mineral surface area, (ii) thermokarst and slumping which expose fine-grained material, and (iii) changes in active layer depth and thickness of the unsaturated zone. All of these could combine in the steep regions of high bedrock exposure where we find the highest *Q*_10_ values (Fig. 5). By modeling increased reactive mineral supply (see Materials and Methods), we can produce the high-temperature sensitivities observed in the Peel catchment (Fig. 6C). They suggest an increase in supply of ~1.5 to 2 times over a 10°C temperature rise, which is broadly consistent with reconstructions of physical weathering and erosion processes in non-glacial landscapes (*16*). These findings indicate that sulfide oxidation can be sensitive to climate not only via reaction kinetics but also dominantly via processes linked to climate change which can increase the supply of reactive surface area.

### Associated CO_2_ flux and projected future change

**Fig. 7.**
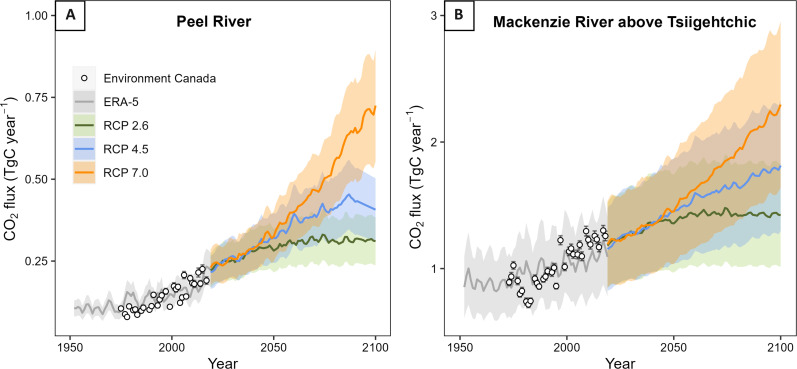
Projections of future CO_2_ fluxes under CMIP6 RCP scenarios. CO_2_ fluxes for (**A**) the Peel River and (**B**) the Mackenzie River mainstem (above Tsiigehtchic) were established from SO_4_^2−^ flux data using estimates of the contribution of SO_4_^2−^ from sulfide oxidation [100% for the Peel River and 82% for the Mackenzie River mainstem (*2*)], and the stoichiometry of Eq. 1. Predictions of SO_4_^2−^ fluxes to 2100 were made by applying the SO_4_^2−^ flux–temperature relationships to projections of catchment-averaged temperature change. The error ranges of CO_2_ fluxes represent the uncertainty on the growth parameter α of the temperature–SO_4_^2−^ flux relationships.

Given the dominant source of SO_4_^2−^ from sulfide oxidation in the Cordillera (*2*, *11*, *25*, *26*, *37*) and its coupling to carbonate weathering (Eq. 1 and fig. S4), we estimate a corresponding CO_2_ release of 1.5 TgC year^−1^ from the MRB (see Materials and Methods). To then assess scenarios of future CO_2_ release via sulfide oxidation, we assume that the observed *Q*_10_ values established over the past few decades (Fig. 4) are representative of future change. This is reasonable if the processes that enhanced SO_4_^2−^ flux (e.g., frost cracking, thermokarst erosion, and hydrological change) and their temperature sensitivity remain prevalent. Projections of air temperature change under a suite of emissions scenarios from the latest Coupled Model Intercomparison Project (CMIP6) are then applied to catchments (see Materials and Methods). Given the current nature of temperature-associated processes in the Mackenzie, we predict that warming under the representative concentration pathway (RCP) 7.0 moderate emission scenario would result in a doubling of the CO_2_ flux associated with sulfide oxidation to 3.0 ± 0.8 TgC year^−1^ by 2100 (Fig. 7).

An increase of ~1.5 TgC to the annual flux of carbon under a moderate emission scenario is equivalent to the CO_2_ generated by Canada’s domestic aviation sector (*68*). To put this in context further, the Taiga Plains ecoregion in the Northwest Territories is estimated to have net ecosystem productivity and CO_2_ drawdown of 5.2 TgC year^−1^ (*69*). At present, human-induced changes to weathering carbon fluxes are not considered in regional and global carbon budget assessments (*70*). Earlier modeling studies concluded that CO_2_ sinks would increase in the MRB with changing weathering patterns (*38*). When assessing trajectories of future warming, it is important to consider the nonlinearity of the temperature sensitivity (Fig. 4), where the exponential response to temperature means that the magnitude of carbon generation is sensitive to the extent of warming. If mitigation of warming is possible, such as under the RCP 4.5 scenario, then the increase in carbon emissions will be dampened to ~0.7 TgC year^−1^ (Fig. 7).

The scale of future CO_2_ fluxes will depend on the hydrological and vegetation response to warming and how these factors affect reaction rates and physical weathering. Arctic runoff is projected to increase under high RCP scenarios (*71*), and increasing precipitation and hydrological connectivity may further enhance sulfide oxidation rates through the more efficient export of reaction products while accelerating thermokarst (*59*). Greening of the Arctic as it warms (*72*) however could inhibit sulfide oxidation within regolith if the soil organic matter offers an O_2_ sink (*73*) and physical weathering processes are subdued. On the basis of the mechanisms that we describe, it is possible that over longer timescales, the sensitivity of weathering to temperature may change as temperatures increase. Experimental work also indicates that sensitivity may decrease as temperatures rise above 0°C due to a transition from frozen to unfrozen pores (*62*). We note, however, that the Peel River catchment with a high-temperature sensitivity is only projected to reach an average annual temperature of 0°C by 2100 under the highest emission scenario.

There are additional impacts of increasing sulfide oxidation on the chemistry and quality of stream and river waters. Oxidation of sulfide minerals can release associated trace metals and iron whose subsequent fate is tied to the redox conditions and the transport of dissolved organic matter (*74*). High concentrations of sulfide-sourced metals may have adverse impacts on aquatic life and water resources in communities in the north, while the fate of iron can cause high turbidity and a marked change in river color (*21*). While outside the scope of this study, understanding how the large increases in SO_4_ flux (Fig. 3) manifest in trace metal concentration change is an important research priority.

The large SO_4_^2−^ flux associated with sulfide weathering from the MRB of 129 Gmol year^−1^ represents ~10% of total global SO_4_^2−^ flux from pyrite oxidation (*8*) while representing only 1.6% of the global drainage area. Across the Arctic, there is overlap in the distributions of sulfides, carbonates, mountainous areas with exposed rock, and ice-rich thermokarst-sensitive landscapes (*25*), suggesting that vast areas may be susceptible to abrupt changes in response to warming. In the Mackenzie (Fig. 2) and other major Arctic watersheds such as the Yukon and Kolyma (*34*, *37*, *75*), increasing SO_4_^2−^ fluxes highlight the operation of the mineral permafrost carbon cycle feedback. Recent evidence shows that sulfide oxidation is more prevalent across the globe than previously thought (*8*), and our findings of accelerating sulfide oxidation with warming in the Mackenzie are consistent with data across high alpine environments in multiple settings (*3*, *9*). The trends here indicate that sulfide oxidation could be a positive feedback on decadal warming and future climate change, but one whose global fluxes and impacts are not well constrained across centennial to longer timescales.

## MATERIALS AND METHODS

### Water chemistry and flux modeling

Point water hydrochemical measurements were obtained by Environment Canada through the National Long-term Water Quality Monitoring Programme (*39*). We analyzed data from 23 sampling sites covering the main stem of the Mackenzie and catchments of and within the Peel, Liard, South Nahanni, Hay, Slave, Peace, Athabasca, Lockhart, and Great Bear (table S1). Dissolved ion concentrations (including SO_4_^2−^ and other major ions, e.g., Ca^2+^ and Mg^2+^) were determined by ion chromatography. Within each year, geochemical data are available at numerous time steps, typically capturing the geochemical signature at both high and low flow regimes. This is important because the hydrograph of the Mackenzie is highly influenced by ice breakup (*32*). To assess this seasonal variation, we defined the seasons as winter (December–April), spring (May–June), summer (July–August), and autumn (September–November). We calculated annual average concentrations for years in which at least one data point was available during periods of low flow (November–April) and high flow (May–October).

Daily water discharge data were obtained from historical records from the Water Survey of Canada (*40*). SO_4_^2−^ flux estimates were calculated using the WRTDS model (*76*) within the EGRET (Exploration and Graphics for RivEr Trends) package (*77*) in R (*78*). WRTDS calculates weighted regressions of paired concentration and discharge data and adjusts the regression by season and time. This was important for assessing decadal trends in the Mackenzie because we see that the relationship between concentration and discharge changes over time in many catchments (Fig. 1B). We use the modified approach of the WRTDS-Kalman Filter (*79*) to account for autocorrelation. Sufficient data (>100 paired water quality and discharge data) were available from 14 sites for WRTDS flux estimates (table S1), and the modeling approach performed well when comparing measured and modeled outputs.

To calculate the Mackenzie-wide SO_4_^2−^ flux, we summed the export from the Mackenzie mainstem above Tsiigehtchic with the export from the Peel River and the Tsiigèhnjik (sites detailed in Fig. 2A). An estimate of the annual average SO_4_^2−^ flux from the Tsiigèhnjik was established using a time series of sampling by the Aurora Institute (fig. S5). Samples were collected from the river surface at 22 time steps from 05 June 2017 to 12 June 2018. Samples were filtered to 0.22 μm and stored in acid-cleaned bottles. Major ions were analyzed using ion chromatography. SO_4_^2−^ flux was calculated using water discharge data from the Water Survey of Canada (*40*). MRB SO_4_^2−^ fluxes and concentrations were compared to datasets from the ArcticGRO dataset (*34*, *80*). Contemporary SO_4_^2−^ concentration in the Mackenzie (~450 μM) is comparable to Arctic catchments of the Yukon and Kolyma, yet is much higher than the Lena, Ob, and Yenisey [ArcticGRO (*80*)] and the world average (*2*, *8*).

Trends in SO_4_^2−^ concentration and flux were established using linear regression, and the statistical significance of change for each catchment was evaluated using the Mann-Kendall nonparametric test in R (*78*). Concentration-discharge relationships were established by comparing water chemistry data and instantaneous discharge. By subsetting by decade, we investigated the change in SO_4_^2−^ concentration for given discharges (Fig. 1B).

### Gridded climate data

Changing water chemistry and water discharge reflect landscape-scale processes occurring across catchments. To assess the temperature sensitivity of weathering reactions in the MRB at a corresponding spatial scale, we used a gridded dataset to assess catchment-specific averages of climate variables. We used the latest reanalysis dataset from ECMWF (ERA5, 0.281° × 0.281°) to produce a monthly time series of air temperature (t2m: temperature at 2 m above the surface) and total precipitation (tp) fields from 1960 to 2020. We created a mask for each catchment using *xy* coordinates of catchment vertexes and used this mask to extract catchment climate variables in Climate Explorer (https://climexp.knmi.nl/start.cgi). The ERA5 reanalysis dataset performed well against historical instrumental records from meteorological stations at sites across the catchment: Fort McPherson (67.43°N, −134.88°E), Inuvik (68.3°N, −133.48°E), Norman Wells (65.28°N, −126.8°E), Hay River (60.83°N, −115.78°E), and Slave Lake (55.3°N, −114.78°E).

To investigate scenarios of future conditions, we used projections of air temperature from the sixth phase of the CMIP. CMIP6 integrates the outputs from a suite of models run with the same experiments (*81*). Here, we interrogate representative concentration pathway (RCP) scenarios 2.6, 4.5, and 7.0, in which RCP 7.0 represents a global net radiative forcing of 7.0 W m^−2^ by 2100 relative to pre-industrial conditions (*82*). These scenarios map onto socioeconomic pathways (*83*). The first of these (RCP 2.6) is an optimistic scenario in which climate protection measures are assumed and a 2°C target is possible, yet the latter (RCP 7.0) represents a moderate emission scenario in which warming globally will likely exceed 2°C. RCP 8.5 exists but has not been used here as it represents a high-end scenario in which no policy-driven mitigation occurs. The CMIP6 dataset provides a gridded output, and mean annual air temperature predictions were extracted for each catchment mask using the same approach as for ERA-5.

### Temperature sensitivity

To investigate the role of temperature on the sulfide oxidation process in the MRB, we examined the relationship between average annual SO_4_^2−^ concentration (for the full suite of rivers) and SO_4_^2−^ flux (for the subset of rivers where sufficient paired concentration and discharge data were available) with mean annual air temperature of each catchment. The relationships are fitted with the exponential model

$$F=F_{0}\cdot \text{exp}\left(\alpha \cdot T\right)$$ (2)

where *F* is the SO_4_^2−^ concentration or flux (in micromolar, kilomoles per year per square kilometer), *F*_0_ is the SO_4_^2−^ concentration or flux at 0°C, *T* is the catchment-averaged temperature (in degree Celsius), and α is the growth rate parameter of the relationship (in reciprocal degree Celsius). We use this parameter to calculate *Q*_10_ values (*48*, *49*) to estimate the change in SO_4_^2−^ export as a result of a 10°C temperature rise

$$Q_{10}=\text{exp}\;\left(10\cdot \alpha \right)$$ (3)

For the catchment average temperature, we tested the sensitivity of this relationship to the period in which temperature was averaged over (e.g., the number of preceding months to years) and found that the best explanatory potential, while still retaining the range of temperature change recorded across the catchment, was reached by averaging the temperature of the preceding 3 years.

### Weathering model

To explore the underlying processes leading to a temperature dependence on sulfide oxidation across the MRB (Fig. 4), we use an empirically calibrated weathering model (*66*, *67*). This has been recently applied to understand sulfide oxidation in mountain catchments, returning a set of parameters informed by river geochemistry data (*52*). The model describes sulfide oxidation flux (*W*; in tonnes per square kilometer per year) as a balance between mineral supply by erosion (ε, in tonnes per square kilometer per year) and reaction kinetics

$$W=X_{m}\cdot \varepsilon \cdot \left\{1-\text{exp}\left[-f\left(T\right)\cdot \left(\frac{K\cdot f\left(q_{w}\right)}{\left(\sigma +1\right)}\right)\cdot {\left(\frac{z}{\varepsilon }\right)}^{\sigma +1}\right]\right\}$$ (4)

where *X*_m_ is the mass fraction of chemically mobile material in the bedrock (unitless), i.e., in this case, the mass fraction of sulfide that is chemically mobile in the bedrock undergoing weathering. In the simplest case, this is the sulfur weight % of bedrock. However, data inversion from river solute chemistry can return an *X*_m_ value that is higher than the total fraction of these mineral phases in the rock, which is often interpreted as the preferential mobility of some phases during weathering (*52*). The lumped parameter $\frac{K\cdot f\left(q_{w}\right)}{\left(\sigma +1\right)}$ (year^−(σ+1)^) describes climatic and lithological factors and *z* considers the length scale of weathering (in tonnes per square kilometer). The exponent (σ + 1) accounts for the decline of weathering rates over time in the weathering zone. Last, the parameter *f* (*T*) describes the temperature dependence as

$$f\left(T\right)=\text{exp}\left(\frac{E_{a}}{R\cdot T_{\text{ref}}}-\frac{E_{a}}{R\cdot T_{\text{react}}}\right)$$ (5)

where *E*_a_ is the apparent activation energy (in kilojoules per mole), *R* is the ideal gas constant (in kilojoules per mole per kelvin), *T*_ref_ is a reference temperature, and *T*_react_ is the temperature at the reaction site (table S3).

The purpose of our model exploration is to examine the processes that could lead to the temperature response that we find across the MRB. To do this, we set parameter values based on the largest empirical dataset from the literature (Taiwan rivers), which are provided by Bufe *et al*. (*52*) (table S3). However, the *E*_a_ value is not well constrained by the data inversion of Bufe *et al*. (*52*), and so instead, we use the experimental insight on sulfide oxidation = 90 kJ mol^−1^ (*47*). To avoid overcomplicating the approach due to a lack of in situ temperature measurements, we assume air temperature as a proxy for the surface ground temperature, but note that this could be offset and dampened in space and time (*3*). We set the erosion rate at 295 tonnes km^−2^ year^−1^ as this is the catchment-averaged erosion rate of the Peel River (*84*).

We then modify the model to include a temperature dependence on the supply of weatherable material—an “Enhanced Supply Model.” We do this by increasing the mass fraction of chemically mobile material in the bedrock, *X*_m_, as a function of temperature. This is conceptualized by a mechanism that can increase the chemical availability of fresh minerals to weathering. This could include the following: (i) physical weathering, which increases fine-grained, high surface area mineral supply, e.g., via the frost cracking mechanism (*85*); (ii) thermokarst geomorphic processes which rapidly expose ground-up mineral surfaces; and (iii) a deepening of the active layer and hydrological changes which allow previously saturated porous material to be accessible to the atmosphere. As discussed in the main text, we cannot isolate these potential drivers. However, we note that field studies have shown that the number of hours in the frost cracking window (−3° to −8°C) linearly scales with sediment production rate (*54*). This provides a link between physical weathering increasing surface mineral area per volume of exposed rock with increasing temperature supported by long-term erosion rate studies (*16*). Thus, to explore how temperature-sensitive physical weathering and hydrological processes could affect predicted sulfide oxidation rates, we modify *X*_m_ so that

$$X_{m}=X_{m255}+\beta \cdot \Delta T\cdot X_{m255}$$ (6)

where β (in reciprocal kelvin) is a variable describing the rate of increase in the mass fraction of available weatherable material, and *X*_m255_ is the initial *X*_m_ value at *T* = 255.15 K that represents the modal winter time temperature. Δ*T* is the change in temperature above this reference temperature (table S3). This model captures a temperature-dependent increase in the supply of chemically mobile reactants by physical weathering, and we set values of β to explore how it may explain the observed temperature sensitivity of sulfide oxidation (β values of 0.1 to 0.3). Our findings provide impetus for future work that seeks to implement how physical weathering processes (such as frost cracking) that can enhance the supply of sulfide minerals could be parameterized into weathering models. We also suggest that non–steady-state model solutions should be explored to consider the time dependence and longevity of these reactions.

### Sulfide oxidation CO_2_ flux and projections of future change

We estimate total fluxes from the MRB by combining results from the Mackenzie mainstem above Tsiigehtchic, the Tsiigèhnjik, and the Peel River. The total SO_4_^2−^ flux is 151.6 Gmol year^−1^ and the flux associated with oxidative weathering of pyrite is estimated to be 129 Gmol year^−1^. This correction takes into account estimates of the contribution of sulfide oxidation to SO_4_^2−^ flux in the Mackenzie mainstem [82% (*2*)] and assumes that all SO_4_^2−^ from the Peel and Tsiigèhnjik is from sulfide oxidation. We then use the stoichiometry of Eq. 1 to estimate the associated current CO_2_ flux of 1.5 TgC year^−1^ from the MRB.

To project future CO_2_ fluxes, we apply the catchment-specific temperature controls for the Peel and Mackenzie to projections of future temperature change from RCP scenarios within CMIP6. When projecting future change based on the temperature sensitivity of sulfide oxidation, we assume that contemporary controls on changing weathering rates remain prevalent in the future. This is appropriate within the scope of this study, but we note that there are a number of uncertainties that could be investigated in further modeling work. To calculate the total MRB flux, we incorporate an estimate of SO_4_^2−^ flux from the Tsiigèhnjik. A decadal time series of water chemistry from the Tsiigèhnjik is not available so it was not possible to establish the SO_4_^2−^-temperature relationship for this catchment. We, therefore, used our assessment of flux from the annual time series (fig. S5) and kept the Tsiigèhnjik CO_2_ flux constant in our projections. We note that this flux is also likely to increase over time but is small (5.1 Gmol year^−1^) compared to the total flux (129 Gmol year^−1^).

### Assessment of water flux-driven change in SO_4_^2−^ flux

Changing precipitation rates and permafrost thaw under warming climates are likely to have implications on hydrological regimes in the Arctic (*71*, *86*). Increased runoff and deeper flow paths would enhance connectivity and infiltration of mineral soils (*28*, *29*), increase thaw depth (*87*), and increase SO_4_^2−^ export. Despite evidence of increasing discharge in parts of the Arctic (*88*, *89*), this is so far not evident in the Mackenzie at the annual timescale in any catchment (fig. S2). Within the period of data availability, we also find no statistically significant relationship between mean annual precipitation (tp; ERA-5) and SO_4_^2−^ flux for the Peel River (*r*^2^ = 0.07; *P* = 0.132) and Mackenzie River above Tsiigehtchic (*r*^2^ = 0.01, *P* = 0.468). SO_4_^2−^ flux estimates integrate variability in river discharge, and the temperature sensitivity of SO_4_^2−^ concentration data (fig. S3) is consistent with SO_4_^2−^ flux findings (Fig. 4), suggesting that the relationships we find are not likely to be driven by changes in water flux.

### Catchment controls

We used geospatial analysis to quantify catchment characteristics. Upstream of each sampling location, watersheds were delineated using the void-filled Shuttle Radar Topography Mission digital elevation model (SRTM DEM; 3 arc sec) and the hydrology toolbox in ArcGIS 10.8.2. Geospatial data include the distributions of bedrock geology and major lithological units (*45*), peatland cover (*41*), average relief from the SRTM DEM, land cover (*42*) (Landsat 30 m), permafrost cover (*43*), and ground ice (*44*) (relict and segregated). We performed a PCA on scaled catchment data and used the location of rivers within the PCA plot to examine variability on baseline SO_4_^2−^ yield and temperature sensitivity (*Q*_10_). To evaluate catchment controls further, we created a correlation matrix between characteristics and metrics of *Q*_10_ and mean SO_4_^2−^ yield. This analysis was undertaken using Corrplot (*90*) in R (*78*).

## Supplementary Materials

### This PDF file includes:

Supplementary Text Figs. S1 to S6 Tables S1 to S3 References
