# Supplementary material for: Temperature sensitivity of the mineral permafrost feedback at the continental scale
Source: Sci Adv. 2024 Oct 9;10(41):eadq4893. doi: 10.1126/sciadv.adq4893 (PMC11463311; doi:10.1126/sciadv.adq4893)
Supplement: Supplementary file 1 — Supplementary Text Figs. S1 to S6 Tables S1 to S3 References [file sciadv.adq4893_sm.pdf]

Science Advances
AAAS

Supplementary Materials for

Temperature sensitivity of the mineral permafrost feedback at the continental scale

Ella V. Walsh *et al.*

Corresponding author: Ella V. Walsh, ella.walsh@uib.no

Sci. Adv. **10**, eadq4893 (2024)
DOI: 10.1126/sciadv.adq4893

This PDF file includes:

Supplementary Text
Figs. S1 to S6
Tables S1 to S3
References

Supplementary Text

Accounting for weathering pathways and SO_4^{2-} source

Alongside mineral sulfide oxidation, riverine SO_4^{2-} can be derived from numerous sources such as atmospheric deposition and dissolution of evaporites (*9, 91*). To investigate this, we interrogated data from the literature and data available within the Environment Canada dataset. First, past assessments of sulfate origin using paired $\delta^{34}S_{SO4}$ and $\delta^{18}O_{SO4}$ (*2, 26, 37*) show that SO_4^{2-} fluxes reflect sedimentary sources of pyrite and gypsum, dominated by sulfide oxidation (*2, 25, 26, 37*) notably in the Canadian Cordillera and Peel Plateau (*11*). Sulfur isotopic tracing shows that ~82% of Mackenzie river SO_4^{2-} is derived from oxidative weathering of pyrite, reaching ~100% in the Peel catchment (*2*). Sources of evaporites have been noted in the Mackenzie River Basin (MRB), particularly in locations in the Hay River, Salt River, and Buffalo River (*33*), but evaporites account for only 2.5% of the basin lithology.

Additional insight comes from the ratios of major ions that show that for catchments that are dominated by carbonate weathering, the acidity driving weathering is dominated by sulfuric acid (Fig. S4). The lithology across the basin is varied and this is demonstrated in the variable contribution of carbonate weathering to the weathering budget. We use the ratio of $[SO_4^{2-}]$ to $[Ca^{2+} + Mg^{2+}]$ as a proxy for the fraction of acidity contributed by sulfuric acid. In the Peel and Mackenzie mainstem, trends of increasing sulfate export map onto increasing contribution from sulfuric acid to carbonate weathering, increasing from 55% to 75% and 55% to 65% respectively from 1980 to 2020. We note that in catchments where sulfide oxidation is low or absent (e.g., Lockhart and Hay Rivers), we find unchanging SO_4^{2-} fluxes. In the Lockhart, the river drains a low sulfide region of the Canadian Shield (*92*) and SO_4^{2-} concentrations are low. In the Hay, where Palaeozoic evaporites such as gypsum are found (*33*), SO_4^{2-} concentrations are higher, but are statistically unchanging over time (Fig. S3).

Finally, input of SO_4^{2-} from rainfall is low and there has been no statistically significant increase in atmospheric deposition of SO_4^{2-} over the monitoring period (*93*). We retrieved wet deposition data from Environment Canada monitoring sites in the Athabasca and Great Bear watersheds (*94*). We found precipitation SO_4^{2-} concentrations had an average of 4 μmol L^{-1} which represents < 1% of Mackenzie mainstem average SO_4^{2-} concentration. Atmospheric deposition is also low in the Yukon (*75*) and is not considered to be important across North America in comparison to the SO_4^{2-} flux in rivers (*9*).

Discussion of hydrological pathways and constraint on weathering processes

The seasonal patterns of changing SO_4^{2-} concentrations through time (Fig. S1) can shed additional light on weathering processes when interpreted alongside SO_4^{2-} fluxes. The rivers with the largest increases in SO_4^{2-} export responded in a similar way: the SO_4^{2-} concentrations increased across all discharges and seasonal periods. The overall SO_4^{2-} fluxes were highest in the spring-summer periods, and these increased through time alongside the autumn-winter fluxes. The difference in the time-dependent increases in SO_4^{2-} concentration and flux between winter (baseflow) and spring (high flow) are subtle (Fig. 3, Fig. S1). However, when we look in more detail at relative changes in SO_4^{2-} concentration, we find an increase occurred in the late summer into autumn period (Fig. S6).

These patterns could reflect geochemical signals of increased weathering coupled with changing hydrological connectivity and dominant flow paths through the landscape (*95*). In the winter, when precipitation accumulates as snow in all catchments, the baseflow is likely derived from waters which have the longest residence times in the catchment (*28*, *60*). The SO_4^{2-} concentration and overall flux exported by these flows has increased (Fig. 3, Fig. S1). Following the break-up of ice in the Mackenzie in spring, high flow within the freshet (*32*) dilutes SO_4^{2-} concentrations (Fig. S1), and flow paths may have less interaction time with mineral soils and rock. However, the SO_4^{2-} fluxes are greatest at this time of year as water discharge is high and these fluxes have increased (Fig. 3). This suggests sulfide weathering is also increasing along shorter, more rapid flow paths. Together, the decadal trends suggest an increase in both the production and export of weathering products throughout the year with warming air temperatures.

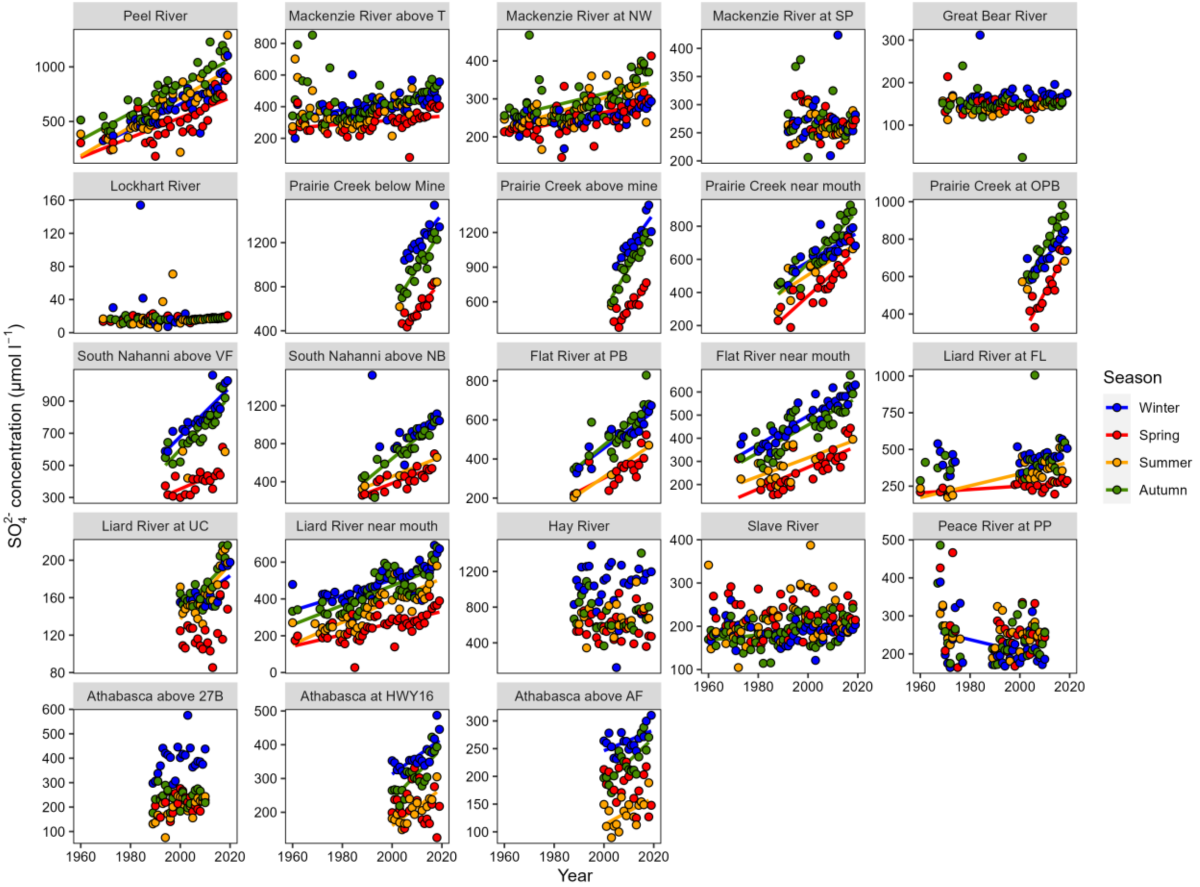

Fig. S1.

Trends in seasonal average of measured SO_4^{2-} concentration by river. See Table S1 for full site names, site locations, and data availability. Significant regression lines have been plotted ($P < 0.05$). Winter: Dec – April, Spring: May – June, Summer: July – Aug, Autumn: Sept – Nov.

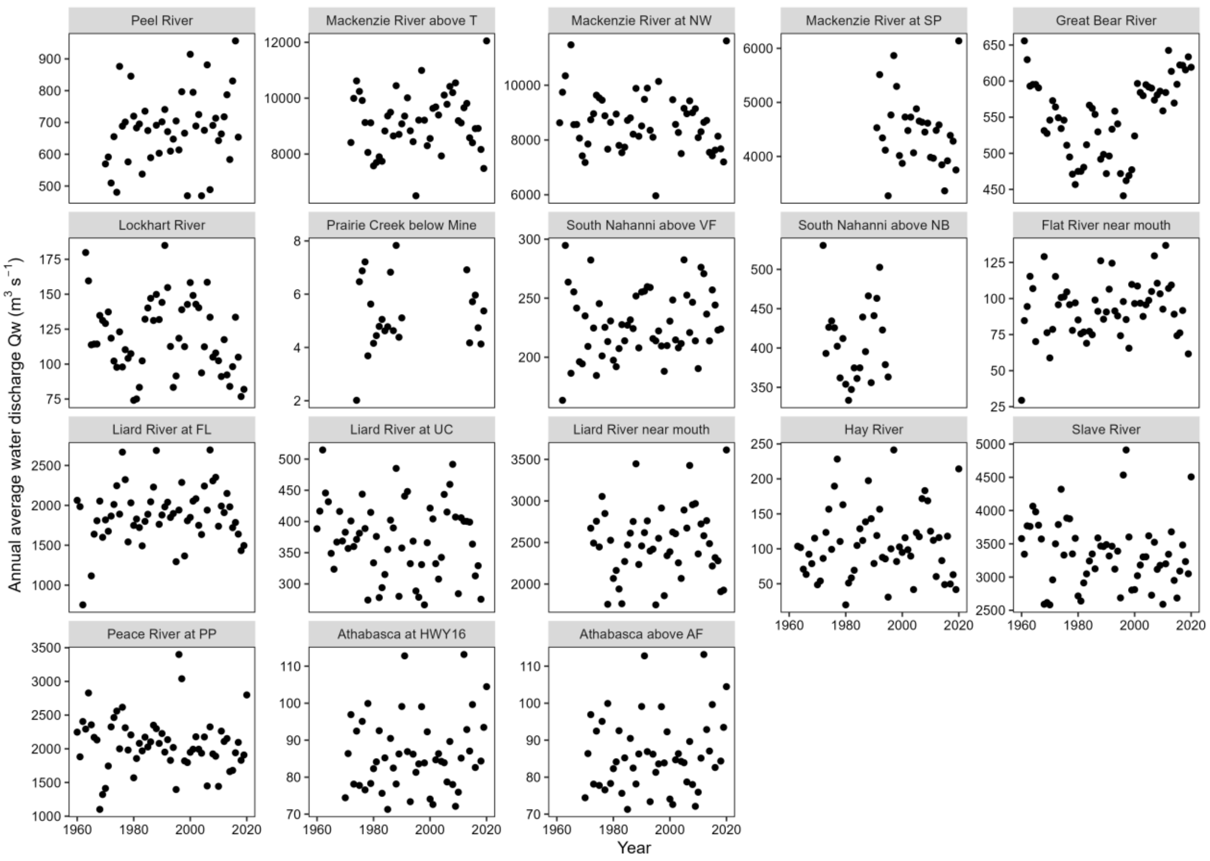

Fig. S2.

Trends in annual averages of measured water discharge by river. No significant change is recorded in discharge over time in any of these rivers.

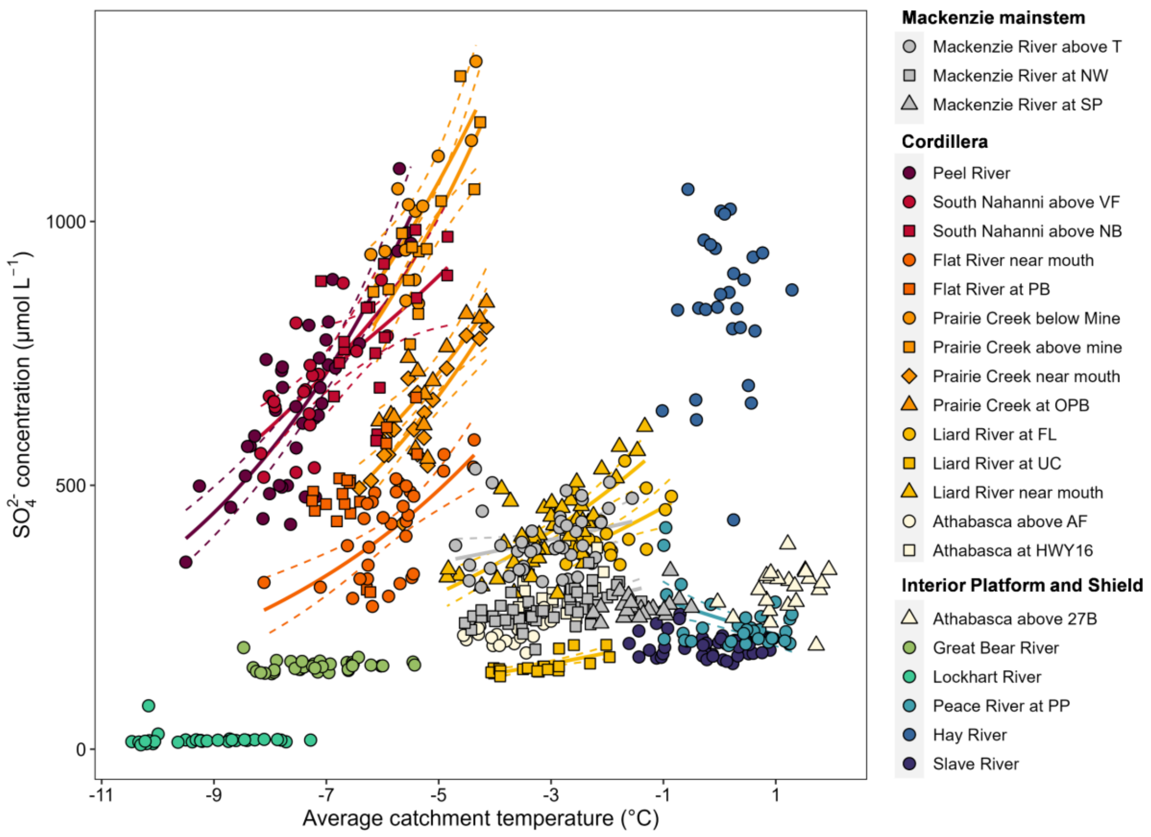

Fig. S3.

Temperature sensitivity of riverine SO_4^{2-} concentration. Exponential function (solid lines) and 95% confidence interval (dashed lines) are drawn for rivers where the relationship is statistically significant ($P < 0.05$). See Table S1 for locations and site codes.

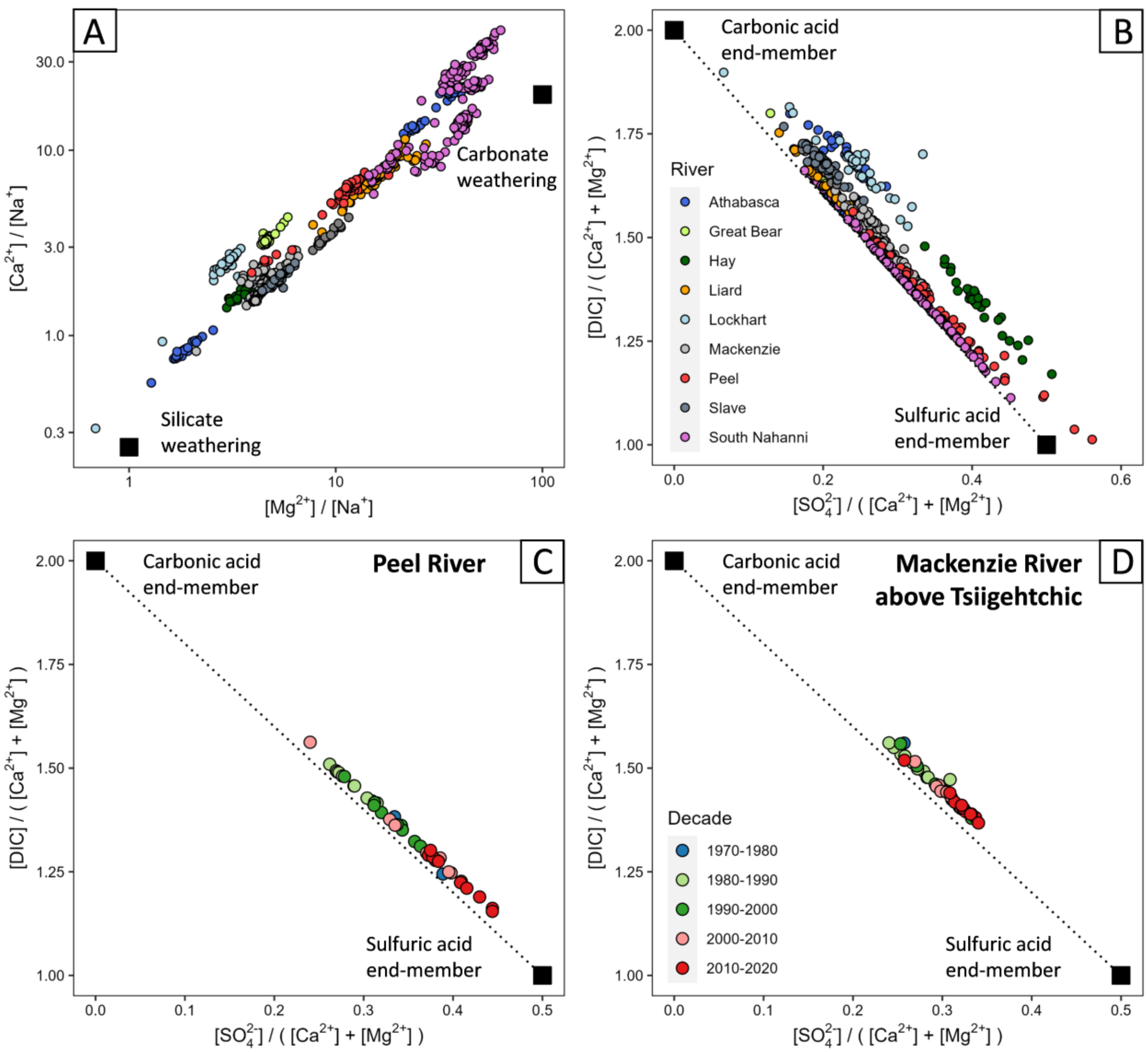

Fig. S4.

Charge balance of Mackenzie River Basin (MRB) weathering pathways in molar ratios. (A) Carbonate – silicate weathering end members for all rivers coloured by major catchment. Each point represents the annual average. **(B)** Carbonic acid – sulfuric acid driven carbonate weathering end members for all rivers. **(C)** and **(D)** Contribution of sulfuric acid to carbonate weathering by decade in the Peel River and the Mackenzie River above Tsiigehtchic.

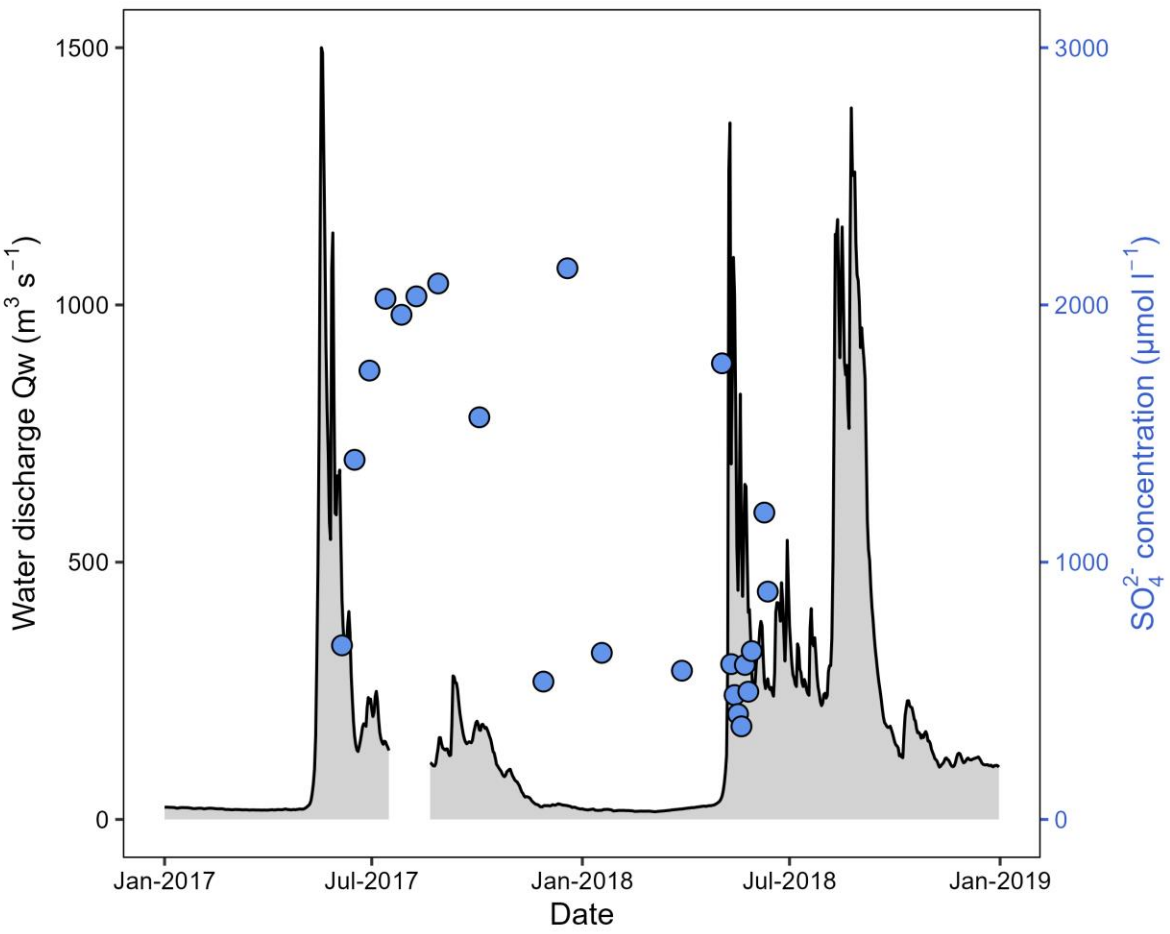

Fig. S5.

Timeseries of Tsiigèhnjik water discharge and SO_4^{2-} concentration data. Samples for geochemical measurements were collected by the Aurora Institute and discharge data was obtained from historical records from the Water Survey of Canada (*40*). Annual average SO_4^{2-} flux: 5.1 Gmol y^{-1}.

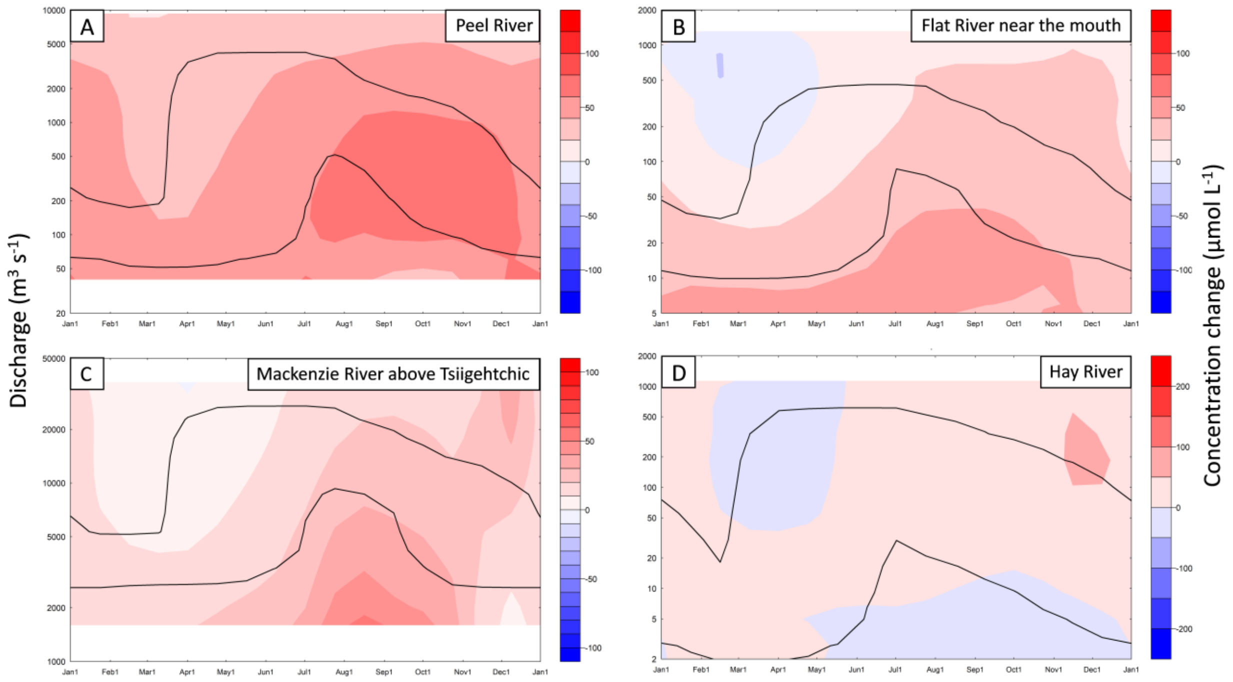

Fig. S6.

Concentration anomaly by month between the start and end of the concentration record. **(A)** Peel River (1970 to 2017), **(B)** Flat River near the mouth (1972 to 2019), **(C)** Mackenzie River above Tsiigehtchic (1972 to 2018), **(D)** Hay River (1988 to 2019). Black lines represent 5th and 95th flow percentiles.

Table S1.

Environment Canada monitoring sites and the timespan for which SO_4^{2-} and water discharge data is available. See Fig. 2A for site locations.

	Site name	Site code	Lat	Long	SO_4^{2-} [a]	Discharge
**1**	Peel River above Fort McPherson	NW10MC0001	67.22	-134.95	1960 - 2019 (39)	1969 - 2017 *
**2**	Mackenzie River above Tsiigehtchic (Arctic Red River)	NW10LA0003	67.46	-133.74	1960 - 2019 (45)	1972 - 2022 *
**3**	Mackenzie River at Norman Wells	NW10KA0001	65.28	-126.85	1960 - 2019 (47)	1943 - 2020 *
**4**	Great Bear River at Great Bear Lake	NW10JC0001	65.14	-123.51	1969 - 2019 (36)	1961 - 2020 *
**5**	Lockhart River at Artillery Lake	NW07RD0001	62.89	-108.47	1969 - 2019 (32)	1944 - 2019 *
**6**	Mackenzie River at Strong Point	NW10FB0006	61.82	-120.79	1992 - 2019 (24)	1991 - 2020 *
**7**	Prairie Creek below Prairie Creek Mine	NW10EC0006	61.55	-124.79	2003 - 2019 (13)	1974 - 2019
**8**	Prairie Creek above Prairie Creek Mine	NW10EC0020	61.61	-124.82	2003 - 2019 (13)	-
**9**	Prairie Creek near the mouth	NW10EC0014	61.28	-124.45	1988 - 2019 (22)	-
**10**	Prairie Creek at the Old Park Boundary	NW10EC0019	61.35	-124.42	2001 - 2019 (14)	-
**11**	South Nahanni River above Virginia Falls	NW10EB0016	61.64	-125.80	1994 - 2019 (20)	1962 - 2018 *
**12**	South Nahanni River above Nahanni Butte	NW10EC0017	61.10	-123.59	1988 - 2019 (17)	1969 - 1995
**13**	Flat River at Park Boundary	NW10EA0008	61.43	-126.63	1988 - 2019 (19)	-
**14**	Flat River near the mouth	NW10EA0004	61.53	-125.41	1972 - 2019 (32)	1960 - 2019 *
**16**	Liard River at Upper Crossing	YT10AA0001	60.05	-128.90	2000 - 2020 (20)	1960 - 2018 *
**15**	Liard River at Fort Liard	NW10ED0001	60.24	-123.48	1960 - 2019 (21)	1942 - 2019
**17**	Liard River near the mouth	NW10ED0002	61.74	-121.22	1960 - 2019 (45)	1972 - 2020 *
**18**	Hay River near Alberta/NWT border	NW07OB0002	60.00	-116.97	1988 - 2019 (28)	1963 - 2020 *
**19**	Slave River at Fitzgerald	AL07NB0001	59.87	-111.59	1960 - 2019 (48)	1921 - 2020 *
**20**	Peace River above Peace Point	AL07KC0001	59.11	-112.43	1967 - 2010 (31)	1960 - 2020 *
**21**	Athabasca River at 27 baseline	AL07DD0001	58.17	-111.37	1989 - 2010 (22)	-
**22**	Athabasca River at HWY 16	AL07AA0023	53.04	-118.09	2000 - 2019 (19)	1913 - 2020 *
**23**	Athabasca River above Athabasca falls	AL07AA0015	52.66	-117.88	2000 - 2019 (19)	1929 - 2020

[a] Number of years for which data is available from both low flow (Nov – April) and high flow (May – Oct) seasons.
* Sufficient paired concentration-discharge data available to undertake WRTDS (Weighted Regressions on Time, Discharge, and Season) flux analysis.

Table S2.

Table of Q_{10} results for SO_4^{2-} yield data. Q_{10} values represent the apparent temperature sensitivity (rate of increase in SO_4^{2-} yield for a 10 °C temperature increase). $Q_{10} = \exp(10\,\alpha)$ using the model $F = F_0 \times \exp(\alpha\, T)$. Total change in flux from start to end of each record. See Methods for further detail.

Site	F_0 (kmol yr^{-1} km^{-2})	α (°C^{-1})	r^2	n	P-value	Q_{10}	Total change (%)
Significant ($p < 0.05$)							
Peel River	932.5 ± 397.1	0.22 ± 0.05	0.34	39	< 0.001	9.15 ± 2.15	166.9
Mackenzie River above Tsiigehtchic	88.9 ± 9.8	0.13 ± 0.04	0.24	45	< 0.001	3.52 ± 1.01	45.2
Mackenzie River at Norman Wells	56.3 ± 3.7	0.08 ± 0.02	0.25	43	< 0.001	2.19 ± 0.60	26.6
Liard River at the mouth	163.9 ± 21.4	0.19 ± 0.04	0.32	47	< 0.001	6.41 ± 1.44	92.6
South Nahanni River at Virginia Falls	2068.2 ± 1084.3	0.26 ± 0.07	0.47	20	< 0.001	13.25 ± 3.45	82.4
Flat River at the mouth	412.0 ± 172.4	0.21 ± 0.06	0.23	40	0.002	7.84 ± 2.42	181.5
Great Bear River	27.4 ± 4.3	0.06 ± 0.02	0.22	31	0.009	1.80 ± 0.67	29.8
Peace River at Peace Point	51.5 ± 1.5	-0.09 ± 0.04	0.13	39	0.027	0.41 ± 0.19	-19.1
Not significant ($p > 0.05$)							
Mackenzie River at Strong Point	32.7 ± 2.9	-0.06 ± 0.06	0.06	25	0.259	0.54 ± -0.51	-13.9
Liard River at Upper Crossing	70.5 ± 10.4	0.07 ± 0.05	0.14	18	0.124	2.01 ± 1.30	6.9
Lockhart River	0.9 ± 0.3	-0.06 ± 0.04	0.04	49	0.199	0.57 ± 0.43	-28.6
Hay River	41.5 ± 3.4	-0.18 ± 0.15	0.05	30	0.238	0.17 ± 0.14	-27.0
Slave River	34.2 ± 0.8	-0.04 ± 0.04	0.03	45	0.283	0.69 ± 0.68	11.8
Athabasca River at HWY16	140.3 ± 37.1	0.11 ± 0.09	0.13	17	0.158	2.94 ± 2.31	56.9

F_0 is the SO_4^{2-} yield at 0 °C.
α is the growth rate parameter of the relationship.
Q_{10} is the change in SO_4^{2-} export as a result of a 10 °C temperature rise.

Table S3.

Variables used in the sulfide weathering model of Bufe et al. (*52*), adapted from West (*66*).

Parameter	Units	Description	Value	Source
W	t km^{-2} yr^{-1}	Weathering yield	Output	
X_m	-	Mass fraction of chemically mobile sulfur in the bedrock	0.05	(*36*)
ε	t km^{-2} yr^{-1}	Erosion yield	295	(*84*)
$f(T)$	-	Temperature sensitivity of reactions	Variable	
E_a	J mol^{-1}	Apparent activation energy	90,000	(*47*)
R	J mol^{-1} K^{-1}	Ideal gas constant	8.3145	
T_{ref}	K	Reference temperature	293.15	(*52*)
T_{react}	K	Temperature at reaction site	Variable	
$\frac{K \cdot f(q_w)}{(\sigma+1)}$	Year $^{-(\sigma+1)}$	Lumped climate and lithological factors	1.3x10^{-4}	(*52*) Taiwan best fit
z	t km^{-2}	Length scale relating to the weathering thickness	4.9x10^{7}	(*52*) Taiwan best fit
σ	-	Describes decline of weathering rates with time in the weathering zone	0.86	(*52*) Taiwan best fit
β	K^{-1}	Describes the increase in supply of chemically mobile phases as a function of changing temperature	Variable	

REFERENCES AND NOTES

1. R. G. Hilton, A. J. West, Mountains, erosion and the carbon cycle. *Nat. Rev. Earth Environ.* **1**, 284–299 (2020).

2. D. Calmels, J. Gaillardet, A. Brenot, C. France-Lanord, Sustained sulfide oxidation by physical erosion processes in the Mackenzie River basin: Climatic perspectives. *Geology* **35**, 1003–1006 (2007).

3. G. Soulet, R. G. Hilton, M. H. Garnett, T. Roylands, S. Klotz, T. Croissant, M. Dellinger, C. Le Bouteiller, Temperature control on CO_2 emissions from the weathering of sedimentary rocks. *Nat. Geosci.* **14**, 665–671 (2021).

4. E. K. Berner, R. A. Berner, *Global Environment: Water, Air, and Geochemical Cycles* (Princeton Univ. Press, ed. 2, 2012).

5. M. A. Torres, A. J. West, G. Li, Sulphide oxidation and carbonate dissolution as a source of CO_2 over geological timescales. *Nature* **507**, 346–349 (2014).

6. P. Maffre, N. L. Swanson-Hysell, Y. Goddéris, Limited carbon cycle response to increased sulfide weathering due to oxygen feedback. *Geophys. Res. Lett.* **48**, e2021GL094589 (2021).

7. A. Bufe, N. Hovius, R. Emberson, J. K. C. Rugenstein, A. Galy, H. J. Hassenruck-Gudipati, J.-M. Chang, Co-variation of silicate, carbonate and sulfide weathering drives CO_2 release with erosion. *Nat. Geosci.* **14**, 211–216 (2021).

8. A. Burke, T. M. Present, G. Paris, E. C. M. Rae, B. H. Sandilands, J. Gaillardet, B. Peucker-Ehrenbrink, W. W. Fischer, J. W. McClelland, R. G. M. Spencer, B. M. Voss, J. F. Adkins, Sulfur isotopes in rivers: Insights into global weathering budgets, pyrite oxidation, and the modern sulfur cycle. *Earth Planet. Sci. Lett.* **496**, 168–177 (2018).

9. J. T. Crawford, E.-L. S. Hinckley, M. I. Litaor, J. Brahney, J. C. Neff, Evidence for accelerated weathering and sulfate export in high alpine environments. *Environ. Res. Lett.* **14**, 124092 (2019).

10. M. A. Torres, N. Moosdorf, J. Hartmann, J. F. Adkins, A. J. West, Glacial weathering, sulfide oxidation, and global carbon cycle feedbacks. *Proc. Natl. Acad. Sci. U.S.A.* **114**, 8716–8721 (2017).

11. J. Spence, K. Telmer, The role of sulfur in chemical weathering and atmospheric CO_2 fluxes: Evidence from major ions, $\delta^{13}C_{DIC}$, and $\delta^{34}S_{SO4}$ in rivers of the Canadian Cordillera. *Geochim. Cosmochim. Acta* **69**, 5441–5458 (2005).

12. T. Roylands, R. G. Hilton, E. L. Mcclymont, M. H. Garnett, G. Soulet, S. Klotz, M. Degler, F. Napoleoni, C. Le Bouteiller, Probing the exchange of CO_2 and O_2 in the shallow critical zone during weathering of marl and black shale. *Earth Surf. Dyn.* **12**, 271–299 (2024).

13. X. Gu, P. J. Heaney, F. D. A. A. Reis, S. L. Brantley, Deep abiotic weathering of pyrite. *Science* **370**, eabb8092 (2020).

14. T. C. Hales, J. J. Roering, Climate-controlled variations in scree production, Southern Alps, New Zealand. *Geology* **33**, 701–704 (2005).

15. T. C. Hales, J. J. Roering, A frost "buzzsaw" mechanism for erosion of the eastern Southern Alps, New Zealand. *Geomorphology* **107**, 241–253 (2009).

16. J. A. Marshall, J. J. Roering, D. G. Gavin, D. E. Granger, Late quaternary climatic controls on erosion rates and geomorphic processes in western Oregon, USA. *Geol. Soc. Am. Bull.* **129**, 715–731 (2017).

17. D. C. Roda-Boluda, T. F. Schildgen, H. Wittmann, S. Tofelde, A. Bufe, J. Prancevic, N. Hovius, Elevation-dependent periglacial and paraglacial processes modulate tectonically-controlled erosion of the western southern alps, New Zealand. *J. Geophys. Res. Earth Surf.* **128**, e2023JF007271 (2023).

18. M. Rantanen, A. Y. Karpechko, A. Lipponen, K. Nordling, O. Hyvärinen, K. Ruosteenoja, T. Vihma, A. Laaksonen, The Arctic has warmed nearly four times faster than the globe since 1979. *Commun. Earth Environ.* **3**, 168 (2022).

19. B. K. Biskaborn, S. L. Smith, J. Noetzli, H. Matthes, G. Vieira, D. A. Streletskiy, P. Schoeneich, V. E. Romanovsky, A. G. Lewkowicz, A. Abramov, M. Allard, J. Boike, W. L. Cable, H. H. Christiansen, R. Delaloye, B. Diekmann, D. Drozdov, B. Etzelmüller, G. Grosse, M. Guglielmin, T. Ingeman-Nielsen, K. Isaksen, M. Ishikawa, M. Johansson, H. Johannsson, A. Joo, D. Kaverin, A. Kholodov, P. Konstantinov, T. Kröger, C. Lambiel, J.-P. Lanckman, D. Luo, G. Malkova, I. Meiklejohn, N. Moskalenko, M. Oliva, M. Phillips, M. Ramos, A. B. K. Sannel, D. Sergeev, C. Seybold, P. Skryabin, A. Vasiliev, Q. Wu, K. Yoshikawa, M. Zheleznyak, H. Lantuit, Permafrost is warming at a global scale. *Nat. Commun.* **10**, 264 (2019).

20. S. L. Smith, H. B. O'Neill, K. Isaksen, J. Noetzli, V. E. Romanovsky, The changing thermal state of permafrost. *Nat. Rev. Earth Environ.* **3**, 10–23 (2022).

21. J. A. O'Donnell, M. P. Carey, J. C. Koch, C. Baughman, K. Hill, C. E. Zimmerman, P. F. Sullivan, R. Dial, T. Lyons, D. J. Cooper, B. A. Poulin, Metal mobilization from thawing permafrost to aquatic ecosystems is driving rusting of Arctic streams. *Commun. Earth Environ.* **5**, 268 (2024).

22. E. A. G. Schuur, A. D. McGuire, C. Schädel, G. Grosse, J. W. Harden, D. J. Hayes, G. Hugelius, C. D. Koven, P. Kuhry, D. M. Lawrence, S. M. Natali, D. Olefeldt, V. E. Romanovsky, K. Schaefer, M. R. Turetsky, C. C. Treat, J. E. Vonk, Climate change and the permafrost carbon feedback. *Nature* **520**, 171–179 (2015).

23. S. V. Kokelj, D. Lacelle, T. C. Lantz, J. Tunnicliffe, L. Malone, I. D. Clark, K. S. Chin, Thawing of massive ground ice in mega slumps drives increases in stream sediment and solute flux across a range of watershed scales. *J. Geophys. Res. Earth Surf* **118**, 681–692 (2013).

24. S. V. Kokelj, M. T. Jorgenson, Advances in thermokarst research. *Permafr. Periglac. Process.* **24**, 108–119 (2013).

25. S. Zolkos, S. E. Tank, S. V. Kokelj, Mineral weathering and the permafrost carbon-climate feedback. *Geophys. Res. Lett.* **45**, 9623–9632 (2018).

26. S. E. Tank, R. G. Striegl, J. W. McClelland, S. V. Kokelj, Multi-decadal increases in dissolved organic carbon and alkalinity flux from the Mackenzie drainage basin to the Arctic Ocean. *Environ. Res. Lett.* **11**, 054015 (2016).

27. J. E. Vonk, S. E. Tank, W. B. Bowden, I. Laurion, W. F. Vincent, P. Alekseychik, M. Amyot, M. F. Billet, J. Canário, R. M. Cory, B. N. Deshpande, M. Helbig, M. Jammet, J. Karlsson, J. Larouche, G. Macmillan, M. Rautio, K. M. Walter Anthony, K. P. Wickland, Reviews and syntheses: Effects of permafrost thaw on Arctic aquatic ecosystems. *Biogeosciences* **12**, 7129–7167 (2015).

28. R. F. Connon, W. L. Quinton, J. R. Craig, M. Hayashi, Changing hydrologic connectivity due to permafrost thaw in the lower Liard River valley, NWT, Canada. *Hydrol. Process.* **28**, 4163–4178 (2014).

29. J. C. Koch, Y. Sjöberg, J. A. O'Donnell, M. P. Carey, P. F. Sullivan, A. Terskaia, Sensitivity of headwater streamflow to thawing permafrost and vegetation change in a warming Arctic. *Environ. Res. Lett.* **17**, 044074 (2022).

30. G. Jia, E. Shevliakova, P. Artaxo, N. De Noblet-Ducoudré, R. Houghton, J. House, K. Kitajima, C. Lennard, A. Popp, A. Sirin, R. Sukumar, L. Verchot, Land–climate interactions, in *Climate Change and Land: An IPCC Special Report on Climate Change, Desertification, Land Degradation, Sustainable Land Management, Food Security, and Greenhouse Gas Fluxes in Terrestrial Ecosystems*, P. R. Shukla, J. Skea, E. Calvo Buendia, V. Masson-Delmotte, H.-O. Pörtner, D. C. Roberts, P. Zhai, R. Slade, S. Connors, R. van Diemen, M. Ferrat, E. Haughey, S. Luz, S. Neogi, M. Pathak, J. Petzold, J. Portugal Pereira, P. Vyas, E. Huntley, K. Kissick, B. M, J. Malley, Eds. (Cambridge Univ. Press, 2019), pp. 131–248.

31. R. M. Holmes, J. W. McClelland, B. J. Peterson, S. E. Tank, E. Bulygina, T. I. Eglinton, V. V. Gordeev, T. Y. Gurtovaya, P. A. Raymond, D. J. Repeta, R. Staples, R. G. Striegl, A. V. Zhulidov, S. A. Zimov, Seasonal and annual fluxes of nutrients and organic matter from large rivers to the Arctic ocean and surrounding seas. *Estuaries Coasts* **35**, 369–382 (2012).

32. R. M. Holmes, J. W. McClelland, B. J. Peterson, I. A. Shiklomanov, A. I. Shiklomanov, A. V. Zhulidov, V. V. Gordeev, N. N. Bobrovitskaya, A circumpolar perspective on fluvial sediment flux to the Arctic Ocean. *Global Biogeochem. Cycles* **16**, 45-1–45-14 (2002).

33. R. Millot, J. Gaillardet, B. Dupré, C. J. Allégre, Northern latitude chemical weathering rates: Clues from the Mackenzie river basin, Canada. *Geochim. Cosmochim. Acta* **67**, 1305–1329 (2003).

34. S. E. Tank, J. W. McClelland, R. G. M. Spencer, A. I. Shiklomanov, A. Suslova, F. Moatar, R. M. W. Amon, L. W. Cooper, G. Elias, V. V. Gordeev, C. Guay, T. Y. Gurtovaya, L. S. Kosmenko, E. A. Mutter, B. J. Peterson, B. Peucker-Ehrenbrink, P. A. Raymond, P. F. Schuster, L. Scott, R. Staples, R. G. Striegl, M. Tretiakov, A. V. Zhulidov, N. Zimov, S. Zimov, R. M. Holmes, Recent trends in the chemistry of major northern rivers signal widespread Arctic change. *Nat. Geosci.* **16**, 789–796 (2023).

35. R. G. Hilton, V. Galy, J. Gaillardet, M. Dellinger, C. Bryant, M. O'Regan, D. R. Gröcke, H. Coxall, J. Bouchez, D. Calmels, Erosion of organic carbon in the Arctic as a geological carbon dioxide sink. *Nature* **524**, 84–87 (2015).

36. M. Dellinger, R. G. Hilton, G. M. Nowell, Fractionation of rhenium isotopes in the Mackenzie River basin during oxidative weathering. *Earth Planet. Sci. Lett.* **573**, 117131 (2021).

37. D. R. Van Stempvoort, J. Spoelstra, G. Bickerton, G. Koehler, B. Mayer, M. Nightingale, J. Miller, Sulfate in streams and groundwater in a cold region (Yukon Territory, Canada): Evidence of weathering processes in a changing climate. *Chem. Geol.* **631**, 121510 (2023).

38. E. Beaulieu, Y. Goddëris, Y. Donnadieu, D. Labat, C. Roelandt, High sensitivity of the continental-weathering carbon dioxide sink to future climate change. *Nat. Clim. Chang.* **2**, 346–349 (2012).

39. Environment and Climate Change Canada, *Point Water Hydrochemical Measurements Obtained by Environment Canada* (Environment and Climate Change Canada, 2023); https://open.canada.ca/data/en/dataset/67b44816-9764-4609-ace1-68dc1764e9ea.

40. HYDAT, Environment Canada HYDAT Database National Water Data Archive using the EC Data Explorer, version 2.4, Environment and Climate Change Canada (2023); https://collaboration.cmc.ec.gc.ca/cmc/hydrometrics/www/.

41. J. Xu, P. J. Morris, J. Liu, J. Holden, PEATMAP: Refining estimates of global peatland distribution based on a meta-analysis. *Catena* **160**, 134–140 (2018).

42. R. Latifovic, D. Pouliot, I. Olthof, Circa 2010 land cover of Canada: Local optimization methodology and product development. *Remote Sens.* **9**, 1098 (2017).

43. J. Obu, S. Westermann, A. Bartsch, N. Berdnikov, H. H. Christiansen, A. Dashtseren, R. Delaloye, B. Elberling, B. Etzelmüller, A. Kholodov, A. Khomutov, A. Kääb, M. O. Leibman, A. G. Lewkowicz, S. K. Panda, V. Romanovsky, R. G. Way, A. Westergaard-Nielsen, T. Wu, J. Yamkhin, D. Zou, Northern Hemisphere permafrost map based on TTOP modelling for 2000–2016 at 1 km^2 scale. *Earth Sci. Rev.* **193**, 299–316 (2019).

44. H. B. O'Neill, S. A. Wolfe, C. Duchesne, New ground ice maps for Canada using a paleogeographic modelling approach. *Cryosphere* **13**, 753–773 (2019).

45. J. O. Wheeler, P. F. Hoffman, K. D. Card, A. Davidson, B. V. Sanford, A. V. Okulitch, W. R. Roest, *Geological map of Canada: Natural resources Canada. "A" series map 1860A* (Environment and Climate Change Canada, 1996); https://doi.org/10.4095/208175.

46. E. A. Davidson, I. A. Janssens, Temperature sensitivity of soil carbon decomposition and feedbacks to climate change. *Nature* **440**, 165–173 (2006).

47. R. V. Nicholson, R. W. Gillham, E. J. Rearddn, Pyrite oxidation in carbonate-buffered solution: 1. Experimental kinetics. *Geochim. Cosmochim. Acta* **52**, 1077–1085 (1988).

48. B. Bond-Lamberty, A. Thomson, A global database of soil respiration data. *Biogeosciences* **7**, 1915–1926 (2010).

49. C. E. Hicks Pries, C. Castanha, R. C. Porras, M. S. Torn, The whole-soil carbon flux in response to warming. *Science* **355**, 1420–1423 (2017).

50. K. Horan, R. G. Hilton, M. Dellinger, E. Tipper, V. Galy, D. Calmels, D. Selby, J. Gaillardet, C. J. Ottley, D. R. Parsons, K. W. Burton, Carbon dioxide emissions by rock organic carbon oxidation and the net geochemical carbon budget of the Mackenzie River Basin. *Am. J. Sci.* **319**, 473–499 (2019).

51. M. A. Williamson, J. D. Rimstidt, The kinetics and electrochemical rate-determining step of aqueous pyrite oxidation. *Geochim. Cosmochim. Acta* **58**, 5443–5454 (1994).

52. A. Bufe, J. K. C. Rugenstein, N. Hovius, CO_2 drawdown from weathering is maximized at moderate erosion rates. *Science* **383**, 1075–1080 (2024).

53. R. S. Anderson, Near-surface thermal profiles in alpine bedrock: Implications for the frost weathering of rock weathering of rock. *Arct. Alp. Res.* **30**, 362–372 (1998).

54. R. Delunel, P. A. van der Beek, J. Carcaillet, D. L. Bourlès, P. G. Valla, Frost-cracking control on catchment denudation rates: Insights from in situ produced ^{10}Be concentrations in stream sediments (Ecrins-Pelvoux massif, French Western Alps). *Earth Planet. Sci. Lett.* **293**, 72–83 (2010).

55. R. C. Fletcher, H. L. Buss, S. L. Brantley, A spheroidal weathering model coupling porewater chemistry to soil thicknesses during steady-state denudation. *Earth Planet. Sci. Lett.* **244**, 444–457 (2006).

56. L. Malone, D. Lacelle, S. Kokelj, I. D. Clark, Impacts of hillslope thaw slumps on the geochemistry of permafrost catchments (Stony Creek watershed, NWT, Canada). *Chem. Geol.* **356**, 38–49 (2013).

57. J. Aga, J. Boike, M. Langer, T. Ingeman-Nielsen, S. Westermann, Simulating ice segregation and thaw consolidation in permafrost environments with the CryoGrid community model. *Cryosphere* **17**, 4179–4206 (2023).

58. Z. Fu, Q. Wu, W. Zhang, H. He, L. Wang, Water migration and segregated ice formation in frozen ground: Current advances and future perspectives. *Front Earth Sci.* **10**, 826961 (2022).

59. S. V. Kokelj, J. Tunnicliffe, D. Lacelle, T. C. Lantz, K. S. Chin, R. Fraser, Increased precipitation drives mega slump development and destabilization of ice-rich permafrost terrain, northwestern Canada. *Glob. Planet. Change* **129**, 56–68 (2015).

60. M. J. Winnick, R. W. H. Carroll, K. H. Williams, R. M. Maxwell, W. Dong, K. Maher, Snowmelt controls on concentration-discharge relationships and the balance of oxidative and acid-base weathering fluxes in an alpine catchment, East River, Colorado. *Water Resour. Res.* **53**, 2507–2523 (2017).

61. S. L. Brantley, A. Shaughnessy, M. I. Lebedeva, V. N. Balashov, How temperature-dependent silicate weathering acts as Earth's geological thermostat. *Science* **379**, 382–389 (2023).

62. B. Elberling, Temperature and oxygen control on pyrite oxidation in frozen mine tailings. *Cold Reg. Sci. Technol.* **41**, 121–133 (2005).

63. S. V. Kokelj, T. C. Lantz, J. Tunnicliffe, R. Segal, D. Lacelle, Climate-driven thaw of permafrost preserved glacial landscapes, northwestern Canada. *Geology* **45**, 371–374 (2017).

64. R. A. Segal, T. C. Lantz, S. V. Kokelj, Acceleration of thaw slump activity in glaciated landscapes of the Western Canadian Arctic. *Environ. Res. Lett.* **11**, 034025 (2016).

65. A. Brooker, R. H. Fraser, I. Olthof, S. V. Kokelj, D. Lacelle, Mapping the activity and evolution of retrogressive thaw slumps by tasselled cap trend analysis of a landsat satellite image stack. *Permafr. Periglac. Process.* **25**, 243–256 (2014).

66. A. J. West, Thickness of the chemical weathering zone and implications for erosional and climatic drivers of weathering and for carbon-cycle feedbacks. *Geology* **40**, 811–814 (2012).

67. E. J. Gabet, S. M. Mudd, A theoretical model coupling chemical weathering rates with denudation rates. *Geology* **37**, 151–154 (2009).

68. Environment and Climate Change Canada, *National Inventory Report 1990–2020: Greenhouse gas sources and sinks in Canada* (Environment and Climate Change Canada, 2022); ISSN: 1910-7064. https://publications.gc.ca/collections/collection_2022/eccc/En81-4-2020-3-eng.pdf.

69. Y. Huberman, J. Beckers, R. Brett, G. Castilla, R. Errington, E. C. Fraser-Reid, D. Goodsman, E. H. Hogg, J. Metsaranta, E. Neilson, J. Olesinski, M.-A. Parisien, D. Price, T. Ramsfield, C. Shaw, D. Thompson, M. F. Voicu, E. Whitman, J. Edwards, *The state of the northwest territories forests in the wake of climate change: Baseline conditions and observed changes to forest ecosystems* (Information Report NOR-X-430, Natural Resources Canada, Canadian Forest Service, Northern Forestry Centre, 2022).

70. P. Regnier, L. Resplandy, R. G. Najjar, P. Ciais, The land-to-ocean loops of the global carbon cycle. *Nature* **603**, 401–410 (2022).

71. M. Meredith, M. Sommerkorn, S. Cassotta, C. Derksen, A. Ekaykin, A. Hollowed, G. Kofinas, A. Mackintosh, J. Melbourne-Thomas, M. M. C. Muelbert, G. Ottersen, H. Pritchard, E. A. G. Schuur, Polar regions, in *IPCC Special Report on the Ocean and Cryosphere in a Changing Climate*, H.-O. Pörtner, D. C. Roberts, V. Masson-Delmotte, P. Zhai, M. Tignor, E. Poloczanska, K. Mintenbeck, A. Alegría, M. Nicolai, A. Okem, J. Petzold, B. Rama, N. M. Weyer, Eds. (Cambridge Univ. Press, 2019), pp. 203–320.

72. Z. A. Mekonnen, W. J. Riley, L. T. Berner, N. J. Bouskill, M. S. Torn, G. Iwahana, A. L. Breen, I. H. Myers-Smith, M. G. Criado, Y. Liu, E. S. Euskirchen, S. J. Goetz, M. C. Mack, R. F. Grant, Arctic tundra shrubification: A review of mechanisms and impacts on ecosystem carbon balance. *Environ Res. Lett.* **16**, 053001 (2021).

73. A. K. Tune, J. L. Druhan, C. R. Lawrence, D. M. Rempe, Deep root activity overprints weathering of petrogenic organic carbon in shale. *Earth Planet. Sci. Lett.* **607**, 118048 (2023).

74. C. Hirst, E. Mauclet, A. Monhonval, E. Tihon, J. Ledman, E. A. G. Schuur, S. Opfergelt, Seasonal changes in hydrology and permafrost degradation control mineral element-bound doc transport from permafrost soils to streams. *Global. Biogeochem. Cycles* **36**, e2021GB007105 (2022).

75. R. C. Toohey, N. M. Herman-Mercer, P. F. Schuster, E. A. Mutter, J. C. Koch, Multidecadal increases in the Yukon River Basin of chemical fluxes as indicators of changing flowpaths, groundwater, and permafrost. *Geophys. Res. Lett.* **43**, 12,120–12,130 (2016).

76. R. M. Hirsch, D. L. Moyer, S. A. Archfield, Weighted regressions on time, discharge, and season (WRTDS) with an application to chesapeake Bay River inputs 1. *J. Am. Water Resour. Assoc.* **46**, 857–880 (2010).

77. R. Hirsch, L. DeCicco, J. Murphy, Exploration and Graphics for RivEr Trends (EGRET), version 3.0.9, CRAN (2023); https://cran.r-project.org/web/packages/EGRET/EGRET.pdf.

78. R Core Team, *R: A Language and Environment for Statistical Computing* (R Foundation for Statistical Computing, 2023); www.R-project.org/.

79. Q. Zhang, R. M. Hirsch, River water-quality concentration and flux estimation can be improved by accounting for serial correlation through an autoregressive model. *Water Resour. Res.* **55**, 9705–9723 (2019).

80. J. W. McClelland, S. E. Tank, R. G. M. Spencer, A. I. Shiklomanov, S. Zolkos, R. M. Holmes, Water quality dataset, version 20230707, Arctic Great Rivers Observatory (2023); www.arcticgreatrivers.org/data.

81. V. Eyring, S. Bony, G. A. Meehl, C. A. Senior, B. Stevens, R. J. Stouffer, K. E. Taylor, Overview of the coupled model intercomparison project phase 6 (CMIP6) experimental design and organization. *Geosci. Model Dev.* **9**, 1937–1958 (2016).

82. D. P. van Vuuren, J. Edmonds, M. Kainuma, K. Riahi, A. Thomson, K. Hibbard, G. C. Hurtt, T. Kram, V. Krey, J. F. Lamarque, T. Masui, M. Meinshausen, N. Nakicenovic, S. J. Smith, S. K. Rose, The representative concentration pathways: An overview. *Clim. Change* **109**, 5–31 (2011).

83. B. C. O'Neill, E. Kriegler, K. L. Ebi, E. Kemp-Benedict, K. Riahi, D. S. Rothman, B. J. van Ruijven, D. P. van Vuuren, J. Birkmann, K. Kok, M. Levy, W. Solecki, The roads ahead: Narratives for shared socioeconomic pathways describing world futures in the 21st century. *Glob. Environ. Chang.* **42**, 169–180 (2017).

84. M. A. Carson, J. N. Jasper, F. M. Conly, Magnitude and sources of sediment input to the mackenzie delta, northwest territories, 1974-94. *Arctic* **51**, 116–124 (1998).

85. T. C. Hales, J. J. Roering, Climatic controls on frost cracking and implications for the evolution of bedrock landscapes. *J. Geophys. Res. Earth Surf.* **112**, F02033 (2007).

86. M. A. Walvoord, B. L. Kurylyk, Hydrologic impacts of thawing permafrost–A review. *Vadose Zone J.* **15**, 1–20 (2016).

87. R. Magnússon, A. Hamm, S. V. Karsanaev, J. Limpens, D. Kleijn, A. Frampton, T. C. Maximov, M. M. P. D. Heijmans, Extremely wet summer events enhance permafrost thaw for multiple years in Siberian tundra. *Nat. Commun.* **13**, 1556 (2022).

88. R. Ahmed, T. Prowse, Y. Dibike, B. Bonsal, H. O'Neil, Recent trends in freshwater influx to the arctic ocean from four major arctic-draining rivers. *Water* **12**, 1189 (2020).

89. R. M. Holmes, A. I. Shiklomanov, A. Suslova, M. Tretiakov, J. W. McClelland, L. Scott, R. G. M. Spencer, S. E. Tank, River discharge [in "State of the Climate in 2020"]. *Bull. Am. Meteorol. Soc.* **102**, S290–S292 (2021).

90. T. Wei, V. Simko, *R Package Corrplot: Visualization of a Correlation Matrix* (2017).

91. K. E. Relph, E. I. Stevenson, A. V. Turchyn, G. Antler, M. J. Bickle, J. J. Baronas, S. E. Darby, D. R. Parsons, E. T. Tipper, Partitioning riverine sulfate sources using oxygen and sulfur isotopes: Implications for carbon budgets of large rivers. *Earth Planet. Sci. Lett.* **567**, 116957 (2021).

92. E. Beaulieu, Y. Goddéris, D. Labat, C. Roelandt, D. Calmels, J. Gaillardet, Modeling of water-rock interaction in the Mackenzie basin: Competition between sulfuric and carbonic acids. *Chem. Geol.* **289**, 114–123 (2011).

93. W. Aas, A. Mortier, V. Bowersox, R. Cherian, G. Faluvegi, H. Fagerli, J. Hand, Z. Klimont, C. Galy-Lacaux, C. M. B. Lehmann, C. L. Myhre, G. Myhre, D. Olivié, K. Sato, J. Quaas, P. S. P. Rao, M. Schulz, D. Shindell, R. B. Skeie, A. Stein, T. Takemura, S. Tsyro, R. Vet, X. Xu, Global and regional trends of atmospheric sulfur. *Sci. Rep.* **9**, 953 (2019).

94. Environment and Climate Change Canada, *Atmospheric deposition datasets. Environment and Climate Change Canada Data - Monitoring of Atmospheric Precipitation Chemistry*

(Environment and Climate Change Canada, 2021); https://donnees.ec.gc.ca/data/air/monitor/monitoring-of-atmospheric-precipitation-chemistry/major-ions/.

95. R. G. Hilton, J. M. Turowski, M. Winnick, M. Dellinger, P. Schleppi, K. H. Williams, C. R. Lawrence, K. Maher, M. West, A. Hayton, Concentration-discharge relationships of dissolved rhenium in alpine catchments reveal its use as a tracer of oxidative weathering. *Water Resour. Res.* **57**, e2021WR029844 (2021).
